# Supplementary material for: NUFEB: A massively parallel simulator for individual-based modelling of microbial communities
Source: PLoS Comput Biol. 2019 Dec 12;15(12):e1007125. doi: 10.1371/journal.pcbi.1007125 (PMC6932830; doi:10.1371/journal.pcbi.1007125)
Supplement: S1 File — (PDF) [file pcbi.1007125.s001.pdf]

NUFEB: A Massively Parallel Simulator for Individual-based Modelling of Microbial Communities

Supporting Information (SI)

Bowen Li[1,2], Denis Taniguchi[1], Jayathilake Pahala Gedara[3¤a], Valentina Gogulancea[3,4], Rebeca Gonzalez-Cabaleiro[3¤b], Jinju Chen[3], Andrew Stephen McGough[1], Irina Dana Ofiteru[3], Thomas P Curtis[3*], Paolo Zuliani[1,2*]

1 School of Computing, Newcastle University, Newcastle upon Tyne, United Kingdom
2 Interdisciplinary Computing and Complex bioSystems (ICOS) Research Group, Newcastle University, Newcastle upon Tyne, United Kingdom
3 School of Engineering, Newcastle University, Newcastle upon Tyne, United Kingdom
4 Chemical and Biochemical Engineering Department, University Politehnica of Bucharest, Bucharest, Romania

¤a Current Address: Department of Oncology, University of Oxford, Oxford, United Kingdom
¤b Current Address: School of Engineering, University of Glasgow, Glasgow, United Kingdom
* tom.curtis@ncl.ac.uk(TC); paolo.zuliani@ncl.ac.uk(PZ)

Model validation against biofilm benchmark problems BM2 and BM3

Validation is a critical step to ensure that both the software and the model have been built correctly with respect to the real biological system. To validate NUFEB, we refer to the two biofilm benchmark problems BM2 and BM3 proposed by the International Water Association (IWA) task group on biofilm modelling [1, 2]. For each benchmark problem, we compare the NUFEB simulation results with previous models.

Problem BM2 evaluates how fluid dynamics affects mass transfer in a mono-microbial functional group (heterotroph) biofilm system with spatially heterogeneous architectures. To match the reference model [3], we assume the biomass density is uniform throughout the biofilm and the microbes are stationary, i.e, particles motion is not considered. The three-dimensional biofilm geometries are constructed on pre-defined lattice points in order to keep similar spatial properties (e.g, biofilm average height, area enlargement factor) with the other models. Two geometries are created in this way: a strictly flat biofilm and a heterogeneous (wavy) biofilm morphology. For simplicity, in BM2 we ignore all biological processes except the consumption of organic substrate in the biofilm compartment. The chemical process considered is substrate mass transport due to diffusion and advection, generated from the fluid dynamics. Note that the mass transport process in the biofilm region can be slightly affected by advection due to biofilm porosity. This is different from the model described in [3] where the transport process in the solid region is only governed by diffusion.

Table A shows the BM2 results for the NUFEB simulations and other reference models [4, 1]. Note that all the models (including NUFEB) are deterministic. The results are further analysed using Student's t-test to show if the NUFEB and reference results are statistically different from each other. For each biofilm geometry, we investigated the average surface concentration of substrate under different flow velocities $\mathbf{U}_f$. It can be seen that there is a good agreement in the flat biofilm cases (p-values > 13.7%). The results clearly show that the substrate concentration increases as the fluid velocity becomes higher, since fluid transports more substrate to the downstream area as a result of increased advection. The simulation results for heterogeneous biofilm structure show the same trend. However, the average concentrations in the high $\mathbf{U}_f$ are about 10% lower than the mean value of other simulation results (with p-value = 8.7%). This may due to the variance of biofilm morphologies.

The benchmark problem BM3 models a multi-microbial functional groups and multi-substrate biofilm system. Briefly, two microbial functional groups are considered in the biofilm compartment: aerobic autotrophic

nitrifiers that oxidize ammonium $NH_4{}^+$ to nitrate $NO_3{}^-$, and aerobic heterotrophs that use organic substrate as the electron donor and oxygen O_2 as the electron acceptor. Initially, microbes are randomly distributed on the bottom surface and then grow until the biomass reaches a prescribed value ($250\mu m$ biofilm height). The bulk liquid compartment is assumed to be completely mixed and fluid flow is not taken into account. Therefore, nutrient concentration is only governed by diffusion in the boundary layer compartment, and by diffusion and reaction in the biofilm. Problem BM3 investigates three different cases including a standard case and two special cases with varying initial ammonia concentration. For each case, we evaluated two key variables at biofilm steady states: the concentration of substrate $C_{\mathrm{S,bulk}}$ and ammonia $C_{\mathrm{N,bulk}}$ in bulk liquid. Table B summarises the comparative results between NUFEB and previous models [2]. Each case is run for five replicates due to stochastic initial microbe distribution and cell division, and the average result is then calculated. The stochastic effects of the results from the biofilm morphology are negligible due to the flat biofilm structure. However, one should notice that the difference can be significant when irregular biofilm structures are formed. The Hotelling's T^2 test for two dependent samples is performed for each case to show the differences between the multivariate means of different results [5]. The results show a good agreement except for $C_{\mathrm{N,bulk}}$ in standard N:COD case which is 10% higher than the mean value (with p-value = 6.8%). This may be expected as the value is sensitive to idiosyncrasies of different models [6].

Table A: BM2 results from NUFEB simulations and other reference models: values are average biofilm surface concentration [kg m^{-3}] of a flat and a wavy biofilm with variation of fluid velocity $\mathbf{U}_f$. All the models (including NUFEB) are deterministic. The results of the reference models are taken from [1] and [4].

Model	Flat:High $\mathbf{U}_f$	Flat:Low $\mathbf{U}_f$	Wave:High $\mathbf{U}_f$	Wave:Low $\mathbf{U}_f$
N3c(3D)	3.82	2.86	2.50	1.48
N2b(2D)	3.83	2.87	2.35	1.41
N2d(2D)	3.99	2.94	3.02	1.38
N1s(1D)	3.83	2.89	2.61	1.60
Mean	3.86	2.89	2.62	1.47
**NUFEB(3D)**	**3.95**	**2.92**	**2.26**	**1.38**
p-values from T-test	0.137	0.19	0.087	0.171

Table B: BM3 results from NUFEB simulations and previous models: values are bulk concentrations of substrate and ammonia in a baseline case, a case with high initial ammonia, and a case with low initial ammonia. Each case in NUFEB is run for five replicates due to the stochastic processes. We take the mean values with the deviation. The results of the reference models are taken from [2] and [6].

Model	Standard Case		High N:COD		Low N:COD	
	$C_{\mathrm{S,bulk}}$ g_{COD}/m^3	$C_{\mathrm{N,bulk}}$ g_{N}/m^3	$C_{\mathrm{S,bulk}}$ g_{COD}/m^3	$C_{\mathrm{N,bulk}}$ g_{N}/m^3	$C_{\mathrm{S,bulk}}$ g_{COD}/m^3	$C_{\mathrm{N,bulk}}$ g_{N}/m^3
W(1D)	5.39	1.59	5.86	18.93	4.39	0.48
M1(1D)	4.84	1.45	5.35	20.26	4.98	0.45
CP(2D)	5.14	1.50	5.45	18.15	5.19	0.44
DN(2D)	5.14	1.74	5.56	20.26	4.66	0.48
iDynoMiCS(3D)	5.23	1.46	5.74	17.3	5.05	0.53
Mean	5.15	1.55	5.59	18.98	4.86	0.48
**NUFEB(3D)**	**5.21**$_{\pm 0.10}$	**1.72**$_{\pm 0.14}$	**5.74**$_{\pm 0.19}$	**18.42**$_{\pm 0.13}$	**5.18**$_{\pm 0.17}$	**0.53**$_{\pm 0.08}$
p-values from T^2 test	0.148		0.471		0.068	

Nutrient mass balance

The nutrient mass balance equation is discretised on a Marker-And-Cell (MAC) uniform grid. The concentration scalar S is defined at the centre of the voxel (cubic grid element), and velocity components U_x, U_y, and U_z are defined at the centres of six surfaces of the voxel (Fig A). The temporal and spatial derivatives of the transport equation are discretised by Forward Euler and Central Finite Differences, respectively. For a given nutrient concentration field at time t, the concentration field at next time step can be calculated using following discretised equation:

$$\frac{S^{n+1}_{i,j,k} - S^{n}_{i,j,k}}{\Delta t} + \overline{U}x_{i,j,k}\frac{S^{n}_{i+1/2,j,k} - S^{n}_{i-1/2,j,k}}{\Delta x} + \overline{U}y_{i,j,k}\frac{S^{n}_{i,j+1/2,k} - S^{n}_{i,j-1/2,k}}{\Delta y} + \overline{U}z_{i,j,k}\frac{S^{n}_{i,j,k+1/2} - S^{n}_{i,j,k-1/2}}{\Delta z}$$
$$= \frac{Jx_{i+1/2,j,k} - Jx_{i-1/2}, j, k}{\Delta x} + \frac{Jy_{i,j+1/2,k} - Jy_{i,j-1/2}, k}{\Delta y} + \frac{Jz_{i,j,k+1/2} - Jz_{i,j,k-1/2}}{\Delta z} + R_{i,j,k},$$

where

$$\overline{U}x_{i,j,k} = \frac{Ux_{i+1/2,j,k} - Ux_{i-1/2,\,j,k}}{2}; \overline{U}y_{i,j,k} = \frac{Uy_{i,j+1/2,k} - Uy_{i,j-1/2,k}}{2}; \overline{U}y_{i,j,k} = \frac{Uy_{i,j+1/2,k} - Uy_{i,j-1/2,k}}{2};$$
$$Jx_{i+1/2,j,k} = D\frac{S^{n}_{i+1,j,k} - S^{n}_{i,j,k}}{\Delta x}; Jy_{i,j+1/2,k} = D\frac{S^{n}_{i,j+1,k} - S^{n}_{i,j,k}}{\Delta y}; Jz_{i,j,k+1/2} = D\frac{S^{n}_{i,j,k+1} - S^{n}_{i,j,k}}{\Delta z};$$
$$S^{n}_{i,j,k} = S(i,j,k,t = n\Delta t); S^{n+1}_{i,j,k} = S(i,j,k,t = (n+1)\Delta t)$$

Here D is the diffusion coefficient of the nutrient and $R_{i,j,k}$ is the nutrient consumption rate at grid (i, j, k).

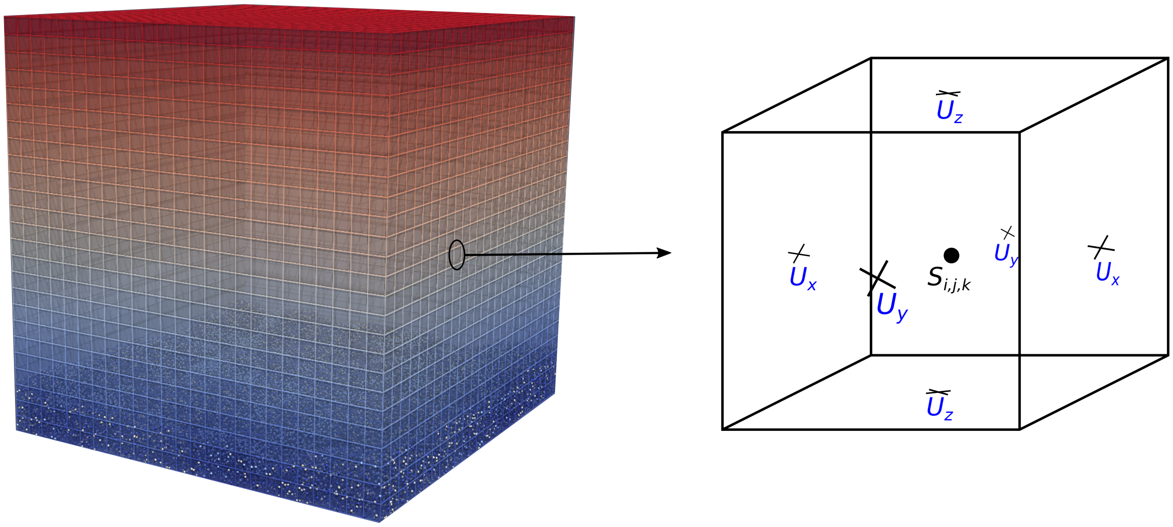

Figure A: **A MAC grid (right)**. Velocity components, U_x, U_y, and U_z, are stored at the centres of six surfaces of the voxel. Nutrient concentration S is stored at the voxel centre.

pH calculations and thermodynamics

An explicit pH calculation module was implemented in NUFEB to handle both hydration reactions (e.g., $CO_2 + H_2O \rightarrow H_2CO_3$) and up to three deprotonations (e.g., $H_2CO_3 \rightarrow HCO_3^- \rightarrow CO_3^{2-}$). The dissociations are assumed to occur instantaneously with respect to the rate of other phenomena considered and are modeled as equilibrium processes. For example, the dissociation reaction for NH_3 is $NH_3 + H_2O \longleftrightarrow {NH_4}^+ + OH^-$. The equilibrium constants (K_{eq}) are computed as:

$$K_{eq} = e^{\frac{-\Delta G_{\text{dissociation}}}{RT}} , \tag{1}$$

where $\Delta G_{\text{dissociation}}$ is the Gibbs free energy corresponding to the dissociation reaction, which is derived from the standard free energies of formation (for example, for the chemical species NH_3, $\Delta G_{f,\text{NH}_3} = -26.57$ and $\Delta G_{f,{\text{NH}_4}^+} = -79.37$ [7]), R is the ideal gas constant (kJ/mol/K), and T is the temperature (K). Moreover, the computation of the dissociation constants and the Gibbs energy of the anabolic and catabolic reactions are corrected for temperature:

$$\Delta G = \Delta G^0 + RTlnQ , \tag{2}$$

where ΔG_r^0 is the standard Gibbs energy, Q is the reaction quotient, R is the ideal gas constant and T is the temperature.

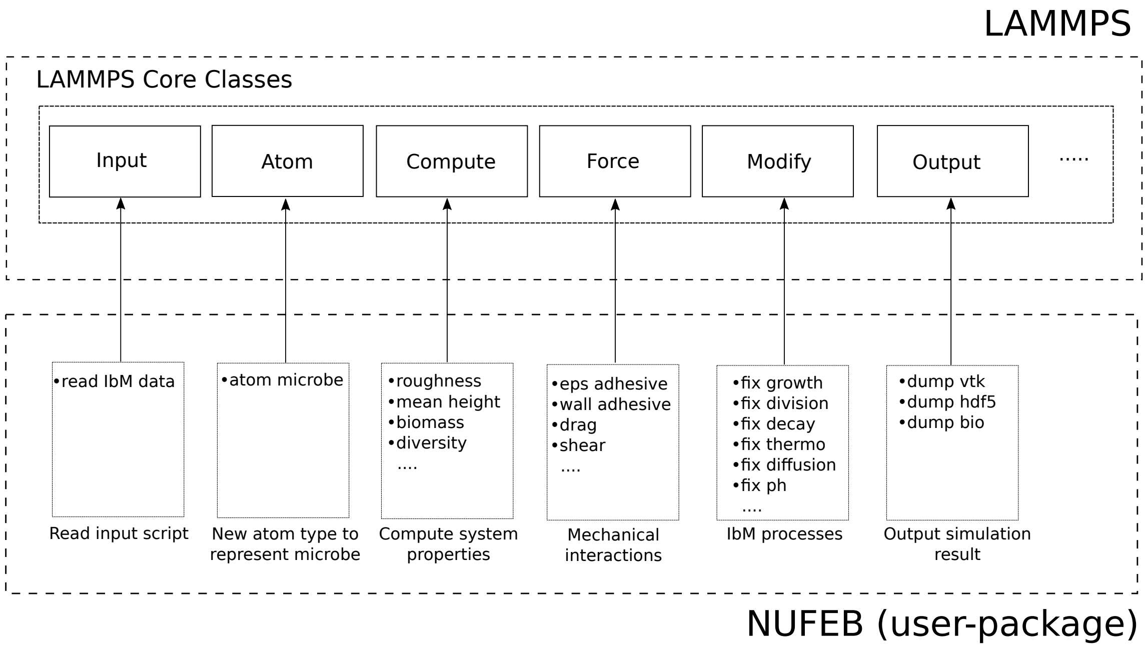

Figure B: **NUFEB tool architecture.** Each small box in the upper level refers to a class in LAMMPS, and each small box in the lower level refers to a collection of classes implemented in NUFEB that inherits from the corresponding parent class in LAMMPS.

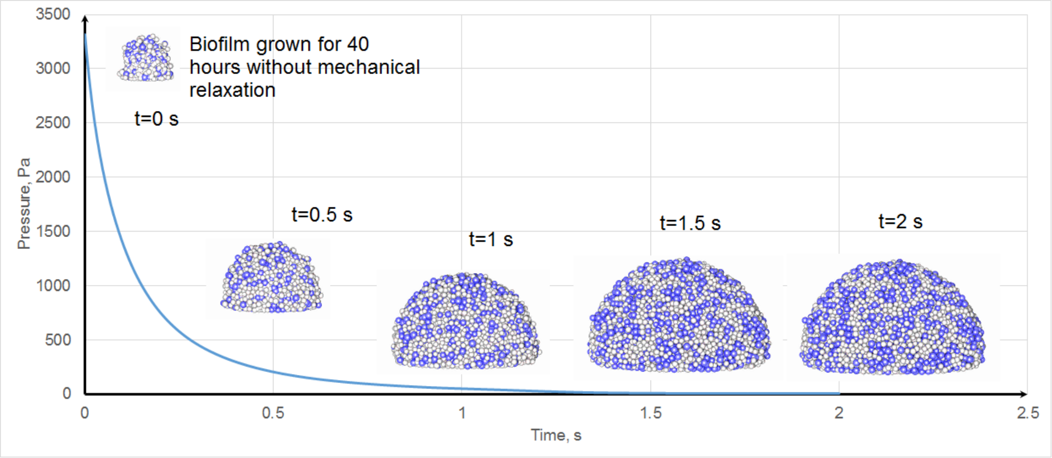

Figure C: **Mechanical relaxation of a biofilm.** The biofilm grows for 40 hours without mechanical relaxation, and then apply the relaxation for 2 seconds. Within 0-1 seconds, the biofilm pressure decreases rapidly and the biofilm shape expands due to the relaxation. The system reaches mechanical equilibrium after 1 second. Correspondingly, the biofilm shape would not change significantly at this stage.

Table C: List of physical parameters used in Case Study 1.

**Parameters**	**Symbol**	**Value**	**Unit**
Domain dimensions	$L_X \times L_Y \times L_Z$	$100 \times 40 \times 100$	μm
Fluid grid cells	$N_X \times N_Y \times N_Z$	$15 \times 6 \times 15$	nodes
Liquid density	ρ_{water}	1000	kg m^{-3}
Kinematic viscosity for water	nu	1×10^{-6}	m^2 s^{-1}
Hamaker coefficient for cohesion	H_a	1×10^{-16}	J
Damping constant for normal contact	γ_n	1×10^6	s$^-$1
Elastic constant for normal contact	K_n	1	N/m
Particle diameter	Dia	1×10^{-6}	m
Particle density	ρ_X	1000	kg m^{-3}

Table D: List of kinetics parameters used in Case Study 2. The parameters for HET and EPS are chosen from [8], and the parameters for AOB and NOB are chosen from [9].

**Parameters**	**Symbol**	**Value**	**Unit**
Heterotroph (HET)			
Maximal specific growth-rate	μ_{H}	6.9×10^{-5}	s
Yield	$\mathrm{Y_H}$	0.61	kg/kg
Decay rate	b_{H}	9.17×10^{-7}	s
Maintenance rate	bm_{H}	3.69×10^{-6}	s
Affinity constant for Substrate	$\mathrm{K_{H,sub}}$	4×10^{-3}	kg m^{-3}
Affinity constant for O_2	$\mathrm{K_{H,O_2}}$	2×10^{-4}	kg m^{-3}
Affinity constant for NO_2	$\mathrm{K_{H,NO_2}}$	3×10^{-4}	kg m^{-3}
Affinity constant for NO_3	$\mathrm{K_{H,NO_3}}$	3×10^{-4}	kg m^{-3}
reduction factor in anoxic condition	η_{H}	0.6	
Ammonia Oxidizer (AOB)			
Maximal specific growth rate	μ_{A}	2.37×10^{-5}	s
Yield	$\mathrm{Y_A}$	0.15	kg/kg
Decay rate	b_{A}	1.27×10^{-6}	s
Maintenance rate	bm_{A}	1.5×10^{-6}	s
Affinity constant for NH_4	$\mathrm{K_{H,NH_4}}$	2.4×10^{-3}	kg m^{-3}
Affinity constant for O_2	$\mathrm{K_{H,O_2}}$	6×10^{-4}	kg m^{-3}
Nitrite Oxidizer (NOB)			
Maximal specific growth-rate	μ_{N}	1.68×10^{-5}	s
Yield	$\mathrm{Y_N}$	0.041	kg/kg
Decay rate	b_{N}	1.27×10^{-6}	s
Maintenance rate	bm_{N}	6.94×10^{-7}	s
Affinity constant for NO_2	$\mathrm{K_{H,NO_2}}$	5.5×10^{-3}	kg m^{-3}
Affinity constant for O_2	$\mathrm{K_{H,O_2}}$	2.2×10^{-3}	kg m^{-3}
EPS			
Yield	$\mathrm{Y_E}$	0.18	kg/kg
Decay rate	b_{E}	1.97×10^{-6}	s
Diffusion coefficient			
Substrate	$\mathrm{D_{Sub}}$	1.16×10^{-9}	m^2 s^{-1}
Oxygen	$\mathrm{D_{O_2}}$	2.3×10^{-9}	m^2 s^{-1}
Ammonium	$\mathrm{D_{NH_4}}$	1.97×10^{-9}	m^2 s^{-1}
Nitrite	$\mathrm{D_{NO_2}}$	1.85×10^{-9}	m^2 s^{-1}
Nitrate	$\mathrm{D_{NO_3}}$	1.85×10^{-9}	m^2 s^{-1}

Table E: List of reactor and physical parameters, and IbM processes used in Case Study 2.

**Parameters**	**Symbol**	**Value**	**Unit**
Computational Domain			
Dimensions	$L_X \times L_Y \times L_Z$	$600 \times 600 \times 200$	μm
Cartesian grid cells	$N_X \times N_Y \times N_Z$	$150 \times 150 \times 50$	nodes
MPI domain decomposition	$P_X \times P_Y \times P_Z$	$10 \times 10 \times 1$	processors
Reactor parameters			
Reactor volume	V	1.25×10^{-3}	m^3
Biofilm surface area	A_F	0.1	m^2
Flow rate	Q	2.31×10^{-7}	m^3 s^{-1}
Boundary layer thickness	L_L	20	μm
Substrate influent concentration	S_{in}^{Sub}	3×10^{-3}	kg COD m^{-3}
Oxygen influent concentration	$S_{in}^{O_2}$	1×10^{-2}	kg m^{-3}
Ammonium influent concentration	$S_{in}^{NH_4}$	2×10^{-2}	kg N m^{-3}
Nitrite influent concentration	$S_{in}^{NO_2}$	0	kg N m^{-3}
Nitrate influent concentration	$S_{in}^{NO_3}$	0	kg N m^{-3}
Physical and system parameters			
Biomass density	ρ_X	32	kg COD m^{-3}
EPS density	ρ_{EPS}	30	kg m^{-3}
Division diameter	Max_{dia}	1.3	μm
Maximum mechanical iterations		3000	
Biological timestep	dt_{bio}	1200	s
IbM processes			
Biological: Monod-based growth, division, EPS production, death			
Chemical: nutrient mass balance			
Physical: contact force, EPS adhesion			

Table F: Stoichiometric matrix for particulate and soluble components implemented in Monod-based growth model. Here Y_i is the yield for microbial functional group i (i = HET, AOB, NOB, EPS and DEAD), μ_i and b_i are the maximum specific growth and decay rates, respectively, η_i is the reduction factor in anoxic conditions, S_j is the concentration of nutrient j (j = Substrate, O_2, NH_4, NO_2, NO_3), $\mathrm{K}_{i,j}$ is the affinity constant between nutrient j and functional group i, bm_i is the maintenance RATE for i, and X is the biomass density

	Process	Soluble					Particulate					Kinetic Expression
		S_{sub}	$S_{\mathrm{O_2}}$	$S_{\mathrm{NH_4}}$	$S_{\mathrm{NO_2}}$	$S_{\mathrm{NO_3}}$	X_{H}	X_{A}	X_{N}	X_{E}	X_{D}	
	Aerobic growth	$-\frac{1}{\mathrm{Y_H}}$	$-\frac{1-\mathrm{Y_H}-\mathrm{Y_E}}{\mathrm{Y_H}}$				1					$\mu_{\mathrm{H}}\frac{S_{\mathrm{sub}}}{\mathrm{K_{H,\ O_2}}+S_{\mathrm{sub}}}\frac{S_{\mathrm{O_2}}}{\mathrm{K_{H,O_2}}+S_{\mathrm{O_2}}}X_{\mathrm{H}}$
	Anoxic growth on NO_2	$-\frac{1}{\mathrm{Y_H}}$				$-\frac{1-\mathrm{Y_H}-\mathrm{Y_E}}{2.86\mathrm{Y_H}}$	1					$\eta_{\mathrm{H}}\mu_{\mathrm{H}}\frac{S_{\mathrm{sub}}}{\mathrm{K_{H,\ sub}}+S_{\mathrm{sub}}}\frac{S_{\mathrm{NO_2}}}{\mathrm{K_{H,NO_2}}+S_{\mathrm{O_2}}}\frac{\mathrm{K_{H,O_2}}}{\mathrm{Ks_{H,O_2}}+S_{\mathrm{O_2}}}X_{\mathrm{H}}$
	Anoxic growth on NO_3	$-\frac{1}{\mathrm{Y_H}}$			$-\frac{1-\mathrm{Y_H}-\mathrm{Y_E}}{1.17\mathrm{Y_H}}$	-1	1					$\eta_{\mathrm{H}}\mu_{\mathrm{H}}\frac{S_{\mathrm{sub}}}{\mathrm{K_{H,sub}}+S_{\mathrm{sub}}}\frac{S_{\mathrm{NO_3}}}{\mathrm{K_{H,NO_3}}+S_{\mathrm{O_2}}}\frac{\mathrm{K_{H,O_2}}}{\mathrm{K_{H,O_2}}+S_{\mathrm{O_2}}}X_{\mathrm{H}}$
**H**ET	Aerobic Maintenance		-1				-1					$bm_{\mathrm{H}}\frac{S_{\mathrm{O_2}}}{\mathrm{K_{H,O_2}}+S_{\mathrm{O_2}}}X_{\mathrm{H}}$
	Anoxic Maintenance on NO_2				$-\frac{1}{1.17}$		-1					$\eta_{\mathrm{H}}bm_{\mathrm{H}}\frac{S_{\mathrm{NO_2}}}{\mathrm{K_{H,NO_2}}+S_{\mathrm{NO_2}}}\frac{\mathrm{K_{H,O_2}}}{\mathrm{K_{H,O_2}}+S_{\mathrm{O_2}}}X_{\mathrm{H}}$
	Anoxic maintenance on NO_3					$-\frac{1}{2.86}$	-1					$\eta_{\mathrm{H}}bm_{\mathrm{H}}\frac{S_{\mathrm{NO_3}}}{\mathrm{K_{H,NO_3}}+S_{\mathrm{NO_3}}}\frac{\mathrm{K_{H,O_2}}}{\mathrm{K_{H,O_2}}+S_{\mathrm{O_2}}}X_{\mathrm{H}}$
	Decay						-1					$b_{\mathrm{H}}X_{\mathrm{H}}$
	Aerobic growth		$-\frac{3.42-\mathrm{Y_A}}{\mathrm{Y_A}}$	$-\frac{1}{\mathrm{Y_A}}$	$\frac{1}{\mathrm{Y_A}}$			1				$\mu_{\mathrm{A}}\frac{S_{\mathrm{NH_4}}}{\mathrm{K_{A,NH_4}}+S_{\mathrm{NH_4}}}\frac{S_{\mathrm{O_2}}}{\mathrm{K_{A,O_2}}+S_{\mathrm{O_2}}}X_{\mathrm{A}}$
**A**OB	Maintenance		-1					-1				$bm_{\mathrm{A}}\frac{S_{\mathrm{O_2}}}{\mathrm{K_{A,O_2}}+S_{\mathrm{O_2}}}X_{\mathrm{A}}$
	Decay							-1				$b_{\mathrm{A}}X_{\mathrm{A}}$
	Aerobic growth		$-\frac{1.15-\mathrm{Y_N}}{\mathrm{Y_N}}$		-$\frac{1}{\mathrm{Y_N}}$	$\frac{1}{\mathrm{Y_N}}$			1			$\mu_{\mathrm{N}}\frac{S_{\mathrm{NO_2}}}{\mathrm{K_{N,NO_2}}+S_{\mathrm{NO_2}}}\frac{S_{\mathrm{O_2}}}{\mathrm{K_{N,O_2}}+S_{\mathrm{O_2}}}X_{\mathrm{N}}$
**N**OB	Maintenance		-1						-1			$bm_{\mathrm{N}}\frac{S_{\mathrm{O_2}}}{\mathrm{K_{N,O_2}}+S_{\mathrm{O_2}}}X_{\mathrm{N}}$
	Decay								-1			$b_{\mathrm{N}}X_{\mathrm{N}}$
**E**PS	Decay	1								-1		$b_{\mathrm{E}}X_{\mathrm{E}}$
**D**EAD	Decay	1									-1	$b_{\mathrm{D}}X_{\mathrm{D}}$

References

[1] Eberl H, van Loosdrecht M, Morgenroth E, Noguera D, Perez J, Picioreanu C, et al. Modelling a spatially heterogeneous biofilm and the bulk fluid: selected results from Benchmark Problem 2 (BM2). Water Science and Technology. 2004;49:155–62. doi:10.2166/wst.2004.0829.

[2] Noguera D, Picioreanu C. Results from the multi-species Benchmark Problem 3 (BM3) using two-dimensional models. Water Science and Technology. 2004;49:169–76. doi:10.2166/wst.2004.0833.

[3] Eberl H, Picioreanu C, Heijnen S, van Loosdrecht M. Three-dimensional numerical study on the correlation of spatial structure, hydrodynamic conditions, and mass transfer and conversion in biofilms. Chemical Engineering Science. 2000;55:6209–6222. doi:10.1016/S0009-2509(00)00169-X.

[4] Wanner O, Eberl H, Morgenroth E, Noguera D, Picioreanu C, Rittmann B, et al. Mathematical modeling of biofilms. vol. 18. IWA Scientific and Technical Report Series; 2006.

[5] Klaus N, Seija S, Hannu O, David ET. Tools for Multivariate Nonparametrics. 2018;.

[6] Lardon L, V Merkey B, Martins S, Dotsch A, Picioreanu C, Kreft JU, et al. iDynoMiCS: next-generation individual-based modelling of biofilms. Environmental Microbiology. 2011;13:2416–34. doi:10.1111/j.1462-2920.2011.02414.x.

[7] Robert P H, Don G W. Perry's chemical engineers' handbook. New York, New York: McGraw-Hill; 2008.

[8] Ofiţeru I, Bellucci M, Picioreanu C, Lavric V, Curtis T. Multi-scale modelling of bioreactor-separator system for wastewater treatment with two-dimensional activated sludge floc dynamics. Water Research. 2013;50. doi:10.1016/j.watres.2013.10.053.

[9] Picioreanu C, Kreft JU, van Loosdrecht M. Particle-based multidimensional multispecies biofilm model. Applied and Environmental Microbiology. 2004;70. doi:10.1128/AFM.70.5.3024-3040.2004.
