# Supplementary material for: NUFEB: A massively parallel simulator for individual-based modelling of microbial communities
Source: PLoS Comput Biol. 2019 Dec 12;15(12):e1007125. doi: 10.1371/journal.pcbi.1007125 (PMC6932830; doi:10.1371/journal.pcbi.1007125)
Supplement: S1 Manual — (PDF) [file pcbi.1007125.s002.pdf]

NUFEB User Manual

Version 3.0

September 16, 2019

Contents

1 Introduction **1**
1.1 NUFEB features 1

2 LAMMPS **2**
2.1 Introduction to LAMMPS 2

3 NUFEB Compilation Instructions **3**
3.1 Pre-compilation instructions 3
3.2 Downloading NUFEB 3
3.3 Compiling NUFEB 4
3.4 Compiling NUFEB with fluid dynamics solver (CFD-DEM) 5
3.5 Running NUFEB 5
3.6 Running NUFEB with fluid dynamics 6
3.7 Add the NUFEB Executable to Your Path (Optional) 6
3.8 Post-processing 6

4 Input Script **7**
4.1 Input script structure 7
4.1.1 Initialization 7
4.1.2 Microbe, nutrient and simulation domain definition 8
4.1.3 Settings 8
4.1.4 Run a simulation 10
4.2 atom_style command 10
4.3 read_data_bio command 12
4.3.1 Atoms section 13
4.3.2 Nutrients section 13
4.3.3 Type Name section 14
4.3.4 Growth Rate section 14
4.3.5 Yield section 15
4.3.6 Consumption Rate section 15
4.3.7 Maintenance section 15
4.3.8 Decay Rate section 16
4.3.9 Electron Donor section 16
4.3.10 Dissipation section 17
4.3.11 Diffusion Coeffs section 17
4.3.12 Mass Transfer Coefficient section 17
4.3.13 Ks section 18
4.3.14 Catabolism Coeffs section 18
4.3.15 Anabolism Coeffs section 18
4.3.16 Decay Coeffs section 19
4.3.17 Nutrient Energy section 19
4.3.18 Type Energy section 20
4.3.19 Charge Number section 20
4.4 fix kinetics command 21
4.5 fix kinetics/growth/monod command 23
4.6 fix kinetics/growth/energy command 24
4.7 fix kinetics/ph command 25
4.8 fix kinetics/thermo command 26

4.9 fix kinetics/diffusion command 27
4.10 divide command 29
4.11 eps_extract command 30
4.12 death command 31
4.13 epsadh command 32
4.14 walladh command 33
4.15 shear command 34
4.16 cfddem command (Sedifoam thirdparty) 35
4.17 compute ntypes command 36
4.18 compute biomass command 36
4.19 compute diameter command 37
4.20 compute dimension command 37
4.21 compute diversity command 38
4.22 compute ave_height command 38
4.23 compute roughness command 39
4.24 compute segregation command 40
4.25 dump bio command 41
4.26 dump grid command 42
4.27 dump bio/hdf5 command 43

5 The NUFEB developer team **44**

A Define a Multi-loops run **45**

1 Introduction

This document provides information on how to download, compile, and start using NUFEB. NUFEB is a three-dimensional, open-source, and massively parallel Individual Based model (IBm) simulator, distributed under the terms of the GNU Public License. The purpose of NUFEB is to offer a flexible and efficient framework for simulating the growth and dynamics of microbial communities at the micro-scale. A comprehensive IbM is implemented in the solver which explicitly models biological, chemical and physical processes, as well as individual cells. The present solver supports parallel computing and the flexibility of extension and customization.

The tool is implemented as a user package within LAMMPS (package USER-NUFEB). LAMMPS is a molecular dynamics simulator offering basic functionalities for Discrete Element Method (DEM) simulations. NUFEB aims to improve those capabilities with the goal to apply it to biological modelling.

1.1 NUFEB features

The list below highlights NUFEB features, with pointers to specific commands which give more details.

Microbes and nutrients
(`atom_style`, `read_data_bio` commands)

- spherical microbes
- microbial species
- nutrients

Biological features
(`fix kinetics/growth/monod`, `fix kinetics/growth/energy`, `fix divide`, `fix eps_extract`, `fix death` commands)

- monod-based growth
- energy-based growth
- cell division
- EPS production
- cell death

Chemical features
(`fix kinetics/ph`, `fix kinetics/thermo` commands)

- pH
- gas-liquid transfer
- thermodynamics

Physical features
(`fix kinetics/diffusion`, `fix epsadh`, `fix cohesive`, `fix walladh`, `fix shear`, `pair_style gran/hooke/history`, `fix wall/gran`, `fix viscous` commands, USER-CFDDEM package)

- nutrient mass balance
- EPS adhesion
- wall adhesion
- cohesive force
- shear force
- contact force
- viscous force
- hydrodynamics

Output
(`dump`, `dump bio`, `dump grid`, `dump bio/hdf5`, `thermo_style`, `compute ntypes`, `compute biomass`, `compute diameter` , `compute dimension`, `compute diversity` , `compute avg_height`, `compute avg_con`, `compute roughness`, `compute segregation` commands)

- output nutrient, pH and energy fields
- output particle information
- compute and output biofilm characteristics

Post-processing

- routines for post-processing are packaged with NUFEB

2 LAMMPS

2.1 Introduction to LAMMPS

LAMMPS solves the motion of every single particle by simply integrating Newton's equations of motion in response to sum of the forces (short or long range based on their interaction with neighbours). At a particular time instance, motion of each particle is collectively solved when subjected to initial or boundary conditions. In order to maintain computational tractability while calculating the interaction forces, LAMMPS maintains a neighbourhood list for each particle which gets updated every so often. These lists are optimized so that local densities and particle overlaps never becomes non-physical. For parallel simulations, LAMMPS spatially partition the domain into smaller sub-domains assigned to each processors. Interprocessor communications are maintained by storing ghost atom interactions with the sub-domain boundaries. LAMMPS development can be helped by two user manuals: User manual and developer manual. The following links will be helpful for the users to get started on LAMMPS:

1. User manual: `http://lammps.sandia.gov/doc/Manual.pdf`
2. Developers guide: `http://lammps.sandia.gov/doc/Developer.pdf`
3. Tutorials: `http://lammps.sandia.gov/tutorials.html`
4. Commands: `http://lammps.sandia.gov/doc/Section_commands.html`
5. Features: `http://lammps.sandia.gov/features.html`

In the present study, lammps5Nov16 version is developed and newer IBm features and capabilities added, this version will be now on referred as NUFEB.

3 NUFEB Compilation Instructions

This section covers instructions on how to compile NUFEB and get started. NUFEB can be compiled and run on almost any Linux or Mac OS. It is emphasized that present NUFEB version 3.0 has been rigorously tested on Ubuntu-14.x, Ubuntu-16.x, Centos-7, and macOS 10.x. At the moment, the simulator does not support Windows. In near future, pre-built executables, binaries or RPMS will be provided to be used on any OS.

3.1 Pre-compilation instructions

Before compiling NUFEB essential features, please make sure you are installed with the following packages depending upon the operating system used:

- gcc/g++ (`https://gcc.gnu.org/`)
- openmpi (`https://www.open-mpi.org/`)
- git (`https://git-scm.com/`)
- cmake

For example, you can run the following commands to install the packages:
On Ubuntu:

```
$ sudo apt-get update
$ sudo apt-get install git-core g++ openmpi-bin openmpi-common libopenmpi-dev cmake
```

On Centos:

```
$ sudo yum update
$ sudo yum install git gcc-c++ openmpi openmpi-devel cmake
```

On MacOS, you need to use Homebrew to install the required libraries:

```
$ ruby -e "$(curl -fsSL https://raw.githubusercontent.com/Homebrew/
install/master/install)" < /dev/null 2> /dev/null
```

Then type the following command to install the libraries:

```
$  brew install gcc open-mpi cmake git
```

3.2 Downloading NUFEB

There are two ways to get the NUFEB software. However, we highly recommend you to use GIT to download the code.

1. If you have GIT installed on your machine, you can use checkout and update commands to get the NUFEB files once and then stay current. To do this, use the clone command to create a local copy of the NUFEB repository with a command:

   ```
   $ git clone --recursive https://github.com/nufeb/NUFEB.git
   ```

Once the command completes, a new directory named "NUFEB" will be created on your machine which contains the latest NUFEB source code, LAMMPS code and thirdparty libraries.

After initial cloning, as bug fixes and new features are added to NUFEB, as listed on Github release page, you can stay up-to-date by typing the following Git commands from within the "NUFEB" directory:

```
$ git checkout master
$ git pull
```

2. You can download source tarball from NUFEB Github repository. Please note that this will not download LAMMPS source code and any other thirdparty library. You will need to download the LAMMPS source code from LAMMPS Github repository (stable_5Nov2016); vtk library from VTK Github repository (version-8.0.0); SediFOAM library from SediFOAM Github repository (microbial-modelling branch); and hdf5 library from HDF5 Github repository (version-1.10.5). Then put the code to corresponding directory in NUFEB.

3.3 Compiling NUFEB

This section gives instruction on compiling NUFEB without fluid dynamics. Before compiling the code, please make sure you have gcc/g++ and openmpi (for parallel run) installed on your system.

You can use the script file `install.sh` provided in `NUFEB/` directory to compile the programme:

```
$ ./install.sh [--options]
```

The `[--options]` is the additional settings of the compilation. The table below lists all available options:

Option	Description
default	mpi, no vtk, no hdf5
`--enable-vtk`	compile with vtk library
`--enable-hdf5`	compile with hdf5 library
`--enable-vtk-hdf5`	compile with both vtk and hdf5 libraries
`--serial`	compile for non-parallel version

Note that in order to compile NUFEB with VTK or HDF5 library, you will need to install the library provided in `thirdparty/` directory, and then compile the NUFEB code with the corresponding argument. For example, to compile NUFEB with VTK library:

```
$ cd thirdparty
$ ./install-vtk
$ cd ..
$ ./install.sh --enable-vtk
```

When finished, you should have an executable “lmp_mpi” or “lmp_serial” in `NUFEB/lammps/src/` directory.

3.4 Compiling NUFEB with fluid dynamics solver (CFD-DEM)

NUFEB employs and extends an exiting CFD-DEM solver SediFoam for the simulation of hydrodynamics.

The current version of SediFoam is supported by OpenFoam 2.3.0, 2.3.x, 2.4.0. You may refer the website `https://openfoamwiki.net/index.php/Installation/Linux/OpenFOAM-2.4.0` for installing OpenFoam 2.4.0 on any Linux distributions. Then use the script `install-sedifoam.sh` in `thirdparty/` directory to compile the code:

```
$ cd thirdparty
$ ./install-sedifoam.sh
```

Note that if you are using Centos, please replace the line "cat Make/options-ubuntu-openmpi » Make/options" with "cat Make/options-linux-openmpi » Make/options" in the `install-sedifoam.sh` file.

3.5 Running NUFEB

Before running a NUFEB simulation, you may need to type the following command to make sure the VTK and/or HDF5 paths are added to $LD_LIBRARY_PATH.

```
$ bash
```

By default, NUFEB runs by reading commands from standard input. Thus if you run the NUFEB executable by itself, e.g.

```
$ mpirun -np 4 lmp_mpi
```

it will simply wait, expecting commands from the keyboard. Note that for parallel environments you also need to specify number of processors by using '-np X' command-line switch.
Typically you should put commands in an input script and use I/O redirection, e.g.

```
$ mpirun -np 4 lmp_mpi -in Inputscript.lammps
```

If NUFEB is compiled as serial version, you can simply pass the input file into the executable "lmp_serial" in order to run a simulation:

```
$ lmp_serial < Inputscript.lammps
```

NUFEB provides several example cases in the `NUFEB/examples/` directory. To run the examples, go to one of the subdirectories and run NUFEB executable by passing in the input file, for example:

```
$ cd NUFEB/examples/biofilm-monod/
$ mpirun -np 4 ../../lammps/src/./lmp_mpi -in Inputscript.lammps
```

After running, there should be output file in the same directory.

3.6 Running NUFEB with fluid dynamics

Running hydrodynamic NUFEB is using lammpFoam executable compiled in Section 3.4. The file is located in `$FOAM_USER_APPBIN` directory.

NUFEB provides several hydrodynamic biofilm cases in `examples/PAPER-SOFTWARE` directory. To run the examples, go to one of the subdirectories and use `Allrun.sh` file:

```
$ cd NUFEB/examples/PAPER-SOFTWARE/c1-deform-fixwall-0.2u
$ ./Allrun.sh
```

3.7 Add the NUFEB Executable to Your Path (Optional)

To make your life easier, you can add the NUFEB executable to your path using the following command from within `NUFEB/lammps/src/` directory:

```
$ export PATH=$PATH:$PWD
```

This addition, however, will only last for the current session. To permanently add it to your path, add the previous line to your ".bashrc" file in your home directory replacing "$PWD" with the path to your `NUFEB/lammps/src/` directory. Once the executable is on your path, there is no need for pointing the path of NUFEB executable when running NUFEB. The simulation can simply be executed as follows:

```
$ mpirun -np 4 lmp_mpi -in Inputscript.lammps
```

3.8 Post-processing

During the course of a simulation, NUFEB will write and save output files describing the current system state.

If you compile the NUFEB with VTK library, results can be in VTK format and readable by Paraview. See `dump custom/vtk` and `dump grid` for more details. The NUFEB online tutorials provide instructions on how to visualise simulation results with Paraview.

NUFEB also provides routines for post-processing plain simulation outputs. The routine, which is located in `NUFEB/post-processing/` directory, supports for reading in and visualizing the output file dumped by `dump custom` command. (Note that the routine does not support to visualize field information at the moment.) In order to use the routine, you will need to have the following software packages:

- POVray (http://www.povray.org/)
- MATLAB (http://uk.mathworks.com/products/matlab/)

To visualize output file, copy the output file produced by `dump custom` to `NUFEB/post-processing/` directory, then change to this directory and execute the "run.sh" script:

```
$ cp output.lammps ../../post-processing/
$ cd ../../post-processing/
$ ./run.sh
```

This script will process the output file to generate a collection of images for each time point as well as a time-lapse video of the simulation in `0_images/` directory.

4 Input Script

In order to execute NUFEB simulation, an input script (text file) is usually prepared with certain commands and parameters list. NUFEB executes by reading those commands and parameters, one line at a time. When the input script ends, NUFEB exits. Each command causes NUFEB to take some actions. It may set an internal variable, read in a file, or run a simulation.

This section explains the commands used for IBm simulation. We will focus on the newer capabilities and commands in NUFEB package. For the pre-existing LAMMPS commands, features and documentation, please refer to the LAMMPS user manual for more details.

4.1 Input script structure

This section describes the structure of a typical NUFEB input script. We will take the scripts in `NUFEB/examples/` directory as examples for the explanation.

A NUFEB input script typically has 4 parts:

- Initialization
- Microbe and simulation domain definition
- Settings
- Run a simulation

4.1.1 Initialization

Example:

```
atom_style   bio
atom_modify  map array sort 1000 5.0e-7
boundary     pp pp ff
newton       off
processors   * * *
comm_modify  vel yes
...
```

Set parameters that need to be defined before microbes are created or read-in from a file. Most of the commands for the initialization are the pre-existing LAMMPS commands:

- `atom_style` command: define what style and attributes of atoms to use in the simulation.
- `atom_modify` commands: modify certain attributes of atoms defined and saved within NUFEB, in addition to what is specified by the atom_style command.
- `boundary` command: set the style of boundaries for the simulation domain in each dimension.
- `newton` command: turn Newton's 3rd law on or off for pairwise and bonded interactions.
- `processors` command: specify how processors are mapped as a regular 3d grid to the global simulation box.

- comm_modify command: sets parameters that affect the inter-processor communication of atom information.

4.1.2 Microbe, nutrient and simulation domain definition

Example:

```
...
read_data_bio atom.in
...
```

In NUFEB, read_data_bio command is used to initialize microbes, nutrients and simulation domain. The command reads in a data file containing information NUFEB needs to run a simulation.

4.1.3 Settings

Example:

```
...
group HET type 1
...
neighbor       5.0e-7 bin
neigh_modify   delay 0 one 5000
pair_style  gran/hooke/history 1.e-4 NULL 1.e-5 NULL 0.0 1

timestep 10

variable EPSdens equal 30

fix fnl all nve/limit 1e-8
fix fv all viscous 1e-5
fix d1 all divide 100 v_EPSdens v_divMass 31231
...
dump id all custom 10 output.lammps id type diameter x y z
...
```

Once initial microbes, nutrients and simulation domain are defined, a variety of settings can be specified: force field, biological processes, chemical processes, output options, etc. The list below is the summary of the commands that can be used for a NUFEB simulation.

Pre-existing LAMMPS commands
The LAMMPS commands list below are commonly used in IbM simulation

- group command: identify a collection of atoms (microbes) as belonging to a group. The group ID can then be used in other commands such as fix, compute, or dump to act on those atoms together.
- lattice command: define a lattice for use by other commands.
- region command: define a geometric region of space in computational domain.

- `create_atoms` command: create atoms on a lattice, or a single atom (or molecule), or a random collection of atoms in a region.
- `neighbor` command: set parameters that affect the building of pairwise neighbor lists.
- `neigh_modify` command: set parameters that affect the building and use of pairwise neighbor lists.
- `timestep` command: set the timestep size for subsequent molecular dynamics simulations (units: s).
- `pair_style gran/hooke/history` command: set formulas for the contact force between two granular particles.
- `variable` command: assign one or more strings to a variable name for evaluation later in the input script or during a simulation.
- `fix` command: set a fix that will be applied to a group of atoms. In LAMMPS, a "fix" is any operation that is applied to the system during timestepping.
- `fix nve/limit` command: perform constant NVE (Number, Volume and Energy) updates of position and velocity for atoms in the group each timestep.
- `fix nve/sphere` command: perform constant NVE integration to update position, velocity, and angular velocity for finite-size spherical particles in the group each timestep.
- `fix viscous` command: add a viscous damping force to atoms in the group that is proportional to the velocity of the atom.
- `fix wall/gran` command: bound the simulation domain of a granular system with a frictional wall.
- `compute` command: define a computation that will be performed on a group of atoms.
- `dump custom` command: dump a snapshot of atom quantities to one or more files every N timesteps.
- `dump custom/vtk` command: dump a snapshot of atom quantities to one or more files every N timesteps in a format readable by the VTK visualization toolkit.
- `thermo` command: compute and print thermodynamic info (e.g. temperature, energy, pressure) on timesteps at the beginning and end of a simulation.
- `thermo_style` command: set the style and content for printing thermodynamic data to the screen and log file.
- `restart` command: write out a binary restart file with the current state of the simulation every so many timesteps.

NUFEB commands

- `fix kinetics` command: set parameters for a variety of kinetics computations.
- `fix kinetics/growth/monod` command: perform microbe growth and decay based on Monod kinetic.
- `fixkinetics/growth/energy` command: perform microbe growth and decay based on metabolic energy.
- `fix divide` command: perform microbe division.
- `fix eps_extract` command: perform EPS (Extracellular Polymeric Substances) production process on HETs (Heterotrophs).
- `fix death` command: perform microbe death process.
- `fix kinetics/ph` command: add the calculations of pH, iron strength and nutrient activity that effect the energy-based growth kinetics.

- `fix kinetics/thermo` command: add thermodynamic and liquid-gas transfer calculations that effect the energy-based growth kinetics.
- `fix kinetics/diffusion` command: solve mass balance of soluble substrates in biofilm and bulk liquid.
- `fix epsadh` command: bound microbes with EPS adhesive force
- `fix walladh` command: impose an adhesive force between wall and the microbes attaching to the wall.
- `fix shear` command: impose an additional shear force for each microbe in the group.
- `compute ntypes` command: define a computation that calculates total number of each species in the system.
- `compute biomass` command: define a computation that calculates total biomass of each species in the system.
- `compute diameter` command: define a computation that calculates floc equivalent diameter.
- `compute dimension` command: define a computation that calculates fractal dimension.
- `compute diversity` command: define a computation that calculates diversity index of all species in the system
- `compute ave_height` command: define a computation that calculates biofilm average height.
- `compute roughness` command: define a computation that calculates biofilm roughness.
- `compute segregation` command: define a computation that calculates biofilm segregation index.
- `dump bio` command: dump microbe, biofilm, floc and kinetics information to one or more files every N timesteps.
- `dump grid` command: dump field information (pH, concentration, energy, etc) to one or more files every N timesteps in a format readable by the VTK visualization toolkit.
- `read_bio_restart` command: read in a previously saved IbM simulation configuration from a restart file.

4.1.4 Run a simulation

Example:

```
...
run  10000
```

A molecular dynamics simulation is run using the `run` command. Also see Appendix A for defining a multi-loop run.

4.2 atom_style command

Syntax

```
atom_style bio
```

- *bio*: atom style for IBm simulation

Description

Define a biological atom style used in IBm simulation. Classical LAMMPS provides different atom types that could be used by user. These are specified in the input script by the command: `atom_style`. Command must be used before a simulation is setup via `read_data_bio` command. A newer `atom_style`, named "bio", is added to increase the number of attribute. For the new atom style, the particles are spheres and each stores a per-particle diameter, mass, outer diameter and outer mass.

4.3 read_data_bio command

Read in a data file containing information NUFEB needs to run a simulation, i.e, microbe, species, nutrient and computation domain. The file can be ASCII text or a gzipped text file (detected by a .gz suffix). The structure of the data file is important, though many settings and sections are optional or can come in any order. A typical example is the data file "atom.in " in `NUFEB/examples/biofilm-monod-low/` directory.

Format of the header of a data file

A data file has a header and a body. The header appears first. The first line of the header is always skipped; it typically contains a description of the file. Lines can have a trailing comment starting with '#' that is ignored. If the line is blank (only whitespace after comment is deleted), it is skipped. If the line contains a header keyword, the corresponding value(s) is read from the line. If it doesn't contain a header keyword, the line begins the body of the file.

Header lines can come in any order. The value(s) are read from the beginning of the line. Thus the keyword atoms should be in a line like "44 atoms"; the keyword ylo yhi should be in a line like "0.0 1.0e-04 ylo yhi". The following list is the headers that are required for running a NUFEB simulation.

- atoms = # of atoms (microbes) in system
- atom types = # of types (microbial species) in system
- nutrients = # of nutrients in system
- xlo xhi = simulation box boundaries in x dimension
- ylo yhi = simulation box boundaries in y dimension
- zlo zhi = simulation box boundaries in z dimension

Example:

```
IBm Simulation

 44   atoms
 5    atom types
 5    nutrients

 0.0  1.0e-04  xlo xhi
 0.0  4.0e-05  ylo yhi
 0.0  1.0e-04  zlo zhi

 ...
```

Format of the body of a data file

The body of the file contains zero or more sections. The first line of a section has only a keyword. The next line is skipped. The remaining lines of the section contain values. The number of lines depends on the section keyword as described below. Zero or more blank lines can be used between sections. Sections can appear in any order, with a few exceptions as noted in the following sections.

These are the section keywords for the body of the file.

- Atoms, Type Name, Growth Rate, Consumption Rate, Yield Coeffs, Maintenance, Decay Rate, Electron Donor, Dissipation.
- Nutrients, Diffusion Coeffs, Mass Transfer Coeffs.
- Ks, Catabolism Coeffs, Anabolism Coeffs, Decay Coeffs, Nutrient Energy, Type Energy, Charge Number.

4.3.1 Atoms section

- one line per atom
- line syntax: atom-ID type-ID inner-diameter density x y z outer-diameter
- unit: diameter (m), density (kg m^{-3})

Example:

```
Atoms

  1 1 1.0e-6 150 0.5e-5 0.5e-5 1e-6 1.2e-6
  2 2 1.0e-6 150 1.5e-5 0.5e-5 1e-6 1.0e-6
```

Define initial atoms (microbes) and types (microbial species) in the system. The Atoms section must appear before all other sections in the data file. The atoms can be listed in any order. For `atom_style bio`, the particles are spheres. atom-ID is used to identify the atom throughout the simulation and in dump files. Normally, it is a unique value from 1 to Natoms for each atom. The type-ID is a 2nd identifier attached to an atom. Normally, it is a number from 1 to N, identifying which species the microbe belongs to. The inner-diameter and outer-diameter specify the inner and outer sizes of a finite-size spherical microbe. Organism such as Heterotroph (HET) excretes Extracellular Polymeric Substances (EPS) which is initially accumulated as a extra shell beyond the particle. The outer-diameter of these species is the initial size of EPS shell which must be greater or equal than the inner-diameter. For the species that do not produce EPS, their outer-diameter should be equal to the inner-diameter. The density is used in conjunction with the particle volume to set the mass of each particle as mass = density × volume. x,y,z specify the (x,y,z) coordinates of atoms. These must be inside the simulation box.

4.3.2 Nutrients section

- one line per nutrient
- line syntax: nutrient-ID nutrient-name nutrient-status=g/l S_{domain} $S_{bc\text{-}xlo}$ $S_{bc\text{-}xhi}$ $S_{bc\text{-}ylo}$ $S_{bc\text{-}yhi}$ $S_{bc\text{-}zlo}$ $S_{bc\text{-}zhi}$
- units: `fix kinetics/growth/energy` = mol L^{-1};
  `fix kinetics/growth/monod` = kg m^{-3}

Example:

```
Nutrients

    1 o2 l 0.002 0.002 0.002 0.002 0.002 0.002 0.002
    2 go2 g 1e-3 1e-3 1e-3 1e-3 1e-3 1e-3 1e-3
```

Define nutrients and their inlet concentrations in the system. The Nutrients section must appear before all other sections except `Atoms` section in the data file. nutrient-ID is used to identify the nutrient throughout the simulation. The nutrient-ID is a 2nd identifier attached to a nutrient. Normally, it is a number from 1 to N. nutrient-name assigns a string to each nutrient which is used to define nutrient attribute parameters in other sections. nutrient-status can be either l (liquid) or g (gas). It is suggested that the nutrient-name of any gas nutrient should start with prefix 'g', e.g, go2 or gco2. S_{domain} defines the inlet concentration of each nutrient within the simulation domain. $S_{bc\text{-}xlo}$ $S_{bc\text{-}xhi}$ $S_{bc\text{-}ylo}$ $S_{bc\text{-}yhi}$ $S_{bc\text{-}zlo}$ $S_{bc\text{-}zhi}$ define the inlet concentrations of each nutrient in six boundary surfaces. The units of concentration depends on the growth command used for the simulation. *S* is considered to be mol L^{-1} if `fix kinetics/growth/energy` command is used, while *S* is in kg m^{-3} if `fix kinetics/growth/monod` is used.

4.3.3 Type Name section

- one line per type
- line syntax: type-ID type-name

Example:

```
Type Name

    1 het
    2 aob
```

Assign a string to each species type defined in `Atoms` section. The type name must be in accordance with the type-ID. Such name is used to define species attribute parameters in other sections.

4.3.4 Growth Rate section

- one line per type
- line syntax: type-name value
- units: s^{-1}

Example:

```
Growth Rate

    het 0.0000695
    aob 0.0000088
```

Define maximum specific growth rate of each species. The Growth Rate section must be defined if `fix kinetics/growth/monod` command is used for the simulation.

4.3.5 Yield section

- one line per type
- line syntax: type-name value
- units: `fix kinetics/growth/energy` = mol mol^{-1}; `fix kinetics/growth/monod` = kg kg^{-1}

Example:

```
Yield

    het 0.61
    aob 0.33
```

Define biomass yield of each species. The Yield section must be defined if `fix kinetics/growth/energy` or `fix kinetics/growth/monod` command is used for the simulation.

4.3.6 Consumption Rate section

- one line per type
- line syntax: type-name value
- units: mol mol^{-1} s^{-1}

Example:

```
Consumption Rate

   aob 0.000283611
   nob 0.000702222
```

Define maximum specific nutrient consumption rate of each species. The Consumption Rate section must be defined if `fix kinetics/growth/energy` command is used for the simulation.

4.3.7 Maintenance section

- one line per type
- line syntax: type-name value
- units: `fix kinetics/growth/energy` = mol mol^{-1} s^{-1}; `fix kinetics/growth/monod` = s^{-1}

Example:

```
Maintenance

   aob 0.000123376
   nob 0.000134422
```

Define maintenance rate of each species. The Maintenance section must be defined if `fix kinetics/growth/energy` or `fix kinetics/growth/monod` command is used for the simulation.

4.3.8 Decay Rate section

- one line per type
- line syntax: type-name value
- units: `fix kinetics/growth/energy` = mol mol^{-1} s^{-1};
  `fix kinetics/growth/monod` = s^{-1}

Example:

```
Decay Rate

   aob 0.000003694
   nob 0.00000127314
```

Define decay rate of each species. The Decay Rate section must be defined if `fix kinetics/growth/energy` or `fix kinetics/growth/monod` command is used for the simulation.

4.3.9 Electron Donor section

- one line per type
- line syntax: type-name value

Example:

```
Electron Donor

    aob 0.9
    nob 2.9
```

Define electron donor of each species. The Electron Donor section must be defined if `fix kinetics/thermo` command is used with the argument *f_yield* = unfix.

4.3.10 Dissipation section

- one line per type
- line syntax: type-name value
- units: kJ mol^{-1}

Example:

```
Dissipation

   aob 3500
   nob 3500
```

Define dissipation constant of each species. The Dissipation section must be defined if `fix kinetics/thermo` command is used with the argument *f_yield* = unfix.

4.3.11 Diffusion Coeffs section

- one line per nutrient
- line syntax: nutrient-name value
- units: m^2 s^{-1}

Example:

```
Diffusion Coeffs

    o2 2e-9
    go2 0
```

Define diffusion coefficient of each nutrient. The Diffusion Coeffs section must be defined if `fix kinetics/diffusion` command is used for the simulation.

4.3.12 Mass Transfer Coefficient section

- one line per nutrient
- line syntax: nutrient-name value
- units: s^{-1}

Example:

```
KLa

    o2 0.0056
    go2 0.0056
```

Define mass transfer coefficient (KLa) of each nutrient. The Mass Transfer Coeffs section must be defined if `fix kinetics/thermo` command is used with the argument *f_reaction*=close.

4.3.13 Ks section

- one line per type
- line syntax: type-name value-1 value-2 ... value-Nnutrient
- units: `fix kinetics/growth/energy` = mol L^{-1};
  `fix kinetics/growth/monod` = kg m^{-3}

Example:

```
Nutrients

    1 nh3 l 0.002 0.002 0.002 0.002 0.002 0.002 0.002
    2 o2 l 2.8e-4 2.8e-4 2.8e-4 2.8e-4 2.8e-4 2.8e-4 2.8e-4

Ks

    aob 1.71e-04 1.88e-05
```

Define half-velocity constants of each species. The Ks section must be defined if `fix kinetics/growth/energy` or `fix kinetics/growth/monod` command is used for the simulation. The order of the Ks values of each species must be consistent with the nutrient IDs defined in the `Nutrients` section. In the above example, Ks_{nh3}=1.71e-04, and Ks_{o2}=1.88e-05. $Ks_{nutrient}$=0 indicates that the nutrient will not be taken into account when solving Monod equation.

4.3.14 Catabolism Coeffs section

- one line per type
- line syntax: type-name value-1 value-2 ... value-Nnutrient

Example:

```
Nutrients

    1 nh3 l 0.002 0.002 0.002 0.002 0.002 0.002 0.002
    2 no2 l 1e-3 1e-3 1e-3 1e-3 1e-3 1e-3 1e-3

Catabolism Coeffs

    aob -1 1
```

Define catabolism coefficients of each species. The section must be defined if `fix kinetics/growth/energy` or `fix kinetics/thermo` command is used for the simulation. The order of the coefficients of each species must be consistent with the nutrient IDs defined in the `Nutrients` section.

4.3.15 Anabolism Coeffs section

- one line per type

- line syntax: type-name value-1 value-2 ... value-Nnutrient

Example:

```
Nutrients

    1 nh3 l 0.002 0.002 0.002 0.002 0.002 0.002 0.002
    2 no2 l 1e-3 1e-3 1e-3 1e-3 1e-3 1e-3 1e-3

Aatabolism Coeffs

    aob -0.9 0.7
```

Define anabolism coefficients of each species. The section must be defined if `fix kinetics/growth/energy` or `fix kinetics/thermo` command is used for the simulation. The order of the coefficients of each species must be consistent with the nutrient IDs defined in the `Nutrients` section.

4.3.16 Decay Coeffs section

- one line per species
- line syntax: species-name value-1 value-2 ... value-Nnutrient

Example:

```
Nutrients

    1 nh3 l 0.002 0.002 0.002 0.002 0.002 0.002 0.002
    2 no2 l 1e-3 1e-3 1e-3 1e-3 1e-3 1e-3 1e-3

Decay Coeffs

    aob -0.9 0.7
```

Define decay coefficients of each species. The section must be defined if `fix kinetics/growth/energy` or `fix kinetics/growth/monod` command is used for the simulation. The order of the coefficients of each species must be consistent with the nutrient IDs defined in the `Nutrients` section.

4.3.17 Nutrient Energy section

- one line per nutrient
- line syntax: nutrient-name not-hydrated-form fully-protonated0form 1st-deprotonated-form 2nd-deprotonated-form 3rd-deprotonated-form form-flag

Example:

```
Nutrient Energy

   nh3 inf -79.37 -26.57 inf inf 3
   o2  inf  16.4   inf   inf inf 2
```

Define Gibbs energy value for each defined nutrient. The section must be defined if `fix kinetics/growth/energy` command is used for the simulation.

4.3.18 Type Energy section

- one line per type
- line syntax: type-name not-hydrated-form fully-protonated-form 1st-deprotonated-form 2nd-deprotonated-form 3rd-deprotonated-form form-flag

Example:

```
Type Energy

   aob inf -67 inf inf inf 2
```

Define Gibbs energy value for each defined type. The section must be defined if `fix kinetics/growth/energy` command is used for the simulation.

4.3.19 Charge Number section

- one line per nutrient
- line syntax: nutrient-name not-hydrated-form fully-protonated-form 1st-deprotonated-form 2nd-deprotonated-form 3rd-deprotonated-form

Example:

```
Charge Number

   nh3 na 1 0 na na
   no2 na 0 -1 na na
```

Define the charge number of ion for each nutrient. The section must be defined if `fix kinetics/ph` command is used for the simulation.

4.4 fix kinetics command

Syntax

```
fix ID group-ID kinetics Nevery nx ny nz v_diffT v_layer
keyword value
```

- *ID* : user-assigned name for the fix
- *group-ID* : ID of the group of microbes to apply the fix to
- *kinetics* : style name of this fix command
- *Nevery* : call kinetics-related functions every this many timesteps
- *nx, ny, nz* : number of grid elements, x, y, z planes
- *v_diffT* : diffusion timestep (s)
- *v_layer* : thickness of boundary layer (m)
- optional keyword = *temp* or *rth* or *gvol* or *rg* or *ph* or *demflag* or *niter*

  temp value = temperature (default: 298.15K)
  rth value = universal gas constant for thermo ($8.3144e^{-3}$ kJ mol^{-1} K^{-1})
  demflag value = 0 or 1 = flag of DEM run (0)
  niter value = maximum # of iterations in kinetics integration (-1)

Required data sections

`Atoms`, `Nutrients`, `Nutrient Energy`* sections

∗ If `fix kinetics/growth/energy` command is used.

Examples

```
variable diffT equal 1e-4
variable layer equal 2e-4

fix k1 all kinetics 100 60 12 30 v_diffT v_bl
fix k1 all kinetics 100 30 6 20 v_diffT v_bl niter 1000
```

Description

Set parameters and perform kinetics integration process. The procedure is shown in Algorithm 1. The integration will invoke several sub-classes to perform different processes.

nx, ny, nz set the number of grid elements in each of the $x, y,$ and z directions, respectively. Note that in NUFEB the x and y directions represent the two horizontal directions and z is the vertical direction. The algorithms used to solve for the nutrient concentration and other chemistry fields require the equal width of each side of each grid. For example, for the simulation domain with $size = 1\text{e}^{-4} \times 5\text{e}^{-5} \times 1\text{e}^{-4}, nx = 20, ny = 10, nz = 20$ is a valid partition.

v_layer defines thickness of boundary layer between the maximum height of biofilm and bulk. This boundary layer forms part of the pure liquid region within the domain, and in this region only diffusion governs the local concentration. Beyond the boundary layer region the liquid is assumed to be well-mixed, and so the solute concentrations are

Algorithm 1 (kinetics integration procedure)

while nutrient mass balance does not reach steady state **do**
    *update pH field and the concentrations of nutrient dissociation forms (`fix kinetics/ph`)
    *update gas-liquid reaction field(`fix kinetics/thermo`)
    *update reaction field(`fix kinetics/growth/energy`)
    ⋄update reaction field (`fix kinetics/growth/monod`)
    *⋄update nutrient concentration field(`fix kinetics/diffusion`)
end while

*perform microbe growth(`fix kinetics/growth/energy`)
⋄perform microbe growth (`fix kinetics/growth/monod`)
*perform buffer pH(`fix kinetics/ph`)
*⋄update bulk concentration(`fix kinetics/diffusion`)
*⋄update boundary layer position

* if `fix kinetics/growth/energy` is applied
⋄ if `fix kinetics/growth/monod` is applied

kept equal to their concentration in an attached bulk compartment. Note that if *v_layer* is less than zero or greater than *zhi*, it is assumed that the boundary layer region is always from biofilm to *zhi*.

temp and *rth* are the optional parameters that will be used for the calculation of Gibb free energy.

demflag is used to manually switch on or off kinetics integration process. The process will be suspended during mechanical integration if *demflag* = 1. You can find more details on how to use this setting in Appendix multi-loops run.

niter sets the maximum number of iterations in diffusion-reaction integration. This is useful if you are solving a non-steady-state system. For example, a diffusion-reaction system without nutrient supplement.

4.5 fix kinetics/growth/monod command

Syntax

```
fix ID group-ID kinetics/growth/monod v_EPSdens v_etaHET
```

- *ID* : user-assigned name for the fix
- *group-ID* : ID of the group of microbes to apply the fix to
- *kinetics/growth/monod* : style name of this fix command
- *v_EPSdens* : EPS density (kg m^{-3})
- *v_etaHET* : reduction factor in anoxic conditions

Required data sections

`Atoms`, `Nutrients`, `Growth Rate`, `Yield Coeffs`, `Maintenance`, `Ks`, `Decay Rate` sections

Examples

```
variable EPSdens equal 30
variable etaHET equal 0.6

fix kgm all kinetics/growth/monod v_EPSdens v_etaHET
```

Description

Perform microbe growth and decay based on basic Monod kinetics. The growth and decay of active microbes and decay of inactive microbe of biomass are calculated using the following growth kinetic equation:

$$\frac{dm_i}{dt} = r_i m_i$$

where m_i is the mass of the particulate microbe and r_i is the specific growth/decay rate. The specific growth rate is determined by Monod kinetic equation and decay is assumed to be the first order. Details of specific growth/decay rates for various processes can be found in (PG Jayathilake *et al. 2017*)[1]

The function implements growth/decay models for five commonly found microbial functional groups: Heterotrophs (HET), Ammonia oxidizing bacteria (AOB), Nitrogen oxidizing bacteria (NOB), Extracellular polymeric substances (EPS) and Dead microbes (DEAD). Other species cannot be defined in the data file if the simulation is using this command. For the active microorganisms HET, AOB and NOB, NUFEB-3.0 supports to define multi-species within the same functional group. The choice of their growth models is determined by the first three characters of the species name defined in `Atoms` section. For example, you can define two species (types) “hetr” and “hety” with different growth rates and yields in data file. The growth/decay process for the two species are based on HET growth model.

[1]P.G. Jayathilake, P. Gupta, B. Li, C. Masden, O. Oyebamiji, R. GonzÃąlez-Cabaleiro, S. Rushton, B. Bridgens, D. Swailes, B. Allen, S. McGough, P. Zuliani, I.D. Ofiteru, D. Wilkinson, J. Chen, T. Curtis *A mechanistic individual-based model of microbial communities* PLOS One, 12 (8) (2017)

The function also calculates consumption rate R for each microbe based on the nutrient concentration of the grid where the microbe belongs to. The results will be used for solving diffusion-reaction equation in `kinetics/diffusion` function.

4.6 fix kinetics/growth/energy command

Syntax

```
fix ID group-ID kinetics/growth/energy v_EPSdens
```

- *ID* : user-assigned name for the fix
- *group-ID* : ID of the group of microbes to apply the fix to
- *kinetics/growth/energy* : style name of this fix command
- *v_EPSdens* : EPS density (kg m^{-3})

Required data sections

`Atoms`, `Nutrients`, `Consumption Rate`, `Yield Coeffs`, `Maintenance`, `Ks`, `Catabolism Coeffs`, `Anabolism Coeffs`, `Decay Rate` sections.

Examples

```
variable EPSdens equal 30

fix kge all kinetics/growth/energy v_EPSdens
```

Description

Perform microbe growth and decay based on metabolic energy. The growth of each bacteria is described by calculating the amount of energy available for its metabolism in each grid element of the reactor.

$$\mu = Y^{max} \cdot (q^{met} - m^{req}) \qquad \text{if } q^{met} > m^{req}$$
$$\mu = 0 \qquad \text{if } q^{met} = m^{req}$$
$$\mu = -k_d \frac{q^{met} - m^{req}}{m^{req}} \qquad \text{if } q^{met} < m^{req}$$

The maximum growth yield Y^{max} can be either dynamic which is calculated using Energy Dissipation Method implemented in `fix kinetics/thermo` command, or static which is defined in `Yield` section. m^{req} is the maintenance rate defined in `Maintenance` section q^{met} is the metabolic rate which depends on the availability of limiting nutrients (in particular, their dissociation forms). k_d is the decay rate defined in `Decay` section. Then, microbial growth is considered only if the cell is harvesting more energy than the necessary to maintain. Otherwise, microbe will maintain or decay linearly with the lack of energy with a constant. The function also calculates consumption rate R for each microbe based on the nutrient concentration of the grid where the microbe belongs to. The results will be used for solving diffusion-reaction equation in `kinetics/diffusion` function.

4.7 fix kinetics/ph command

Syntax

```
fix ID group-ID kinetics/ph
```

- *ID* : user-assigned name for the fix
- *group-ID* : ID of the group of microbes to apply the fix to
- *kinetics/ph* : style name of this fix command
- *fix* or *dynamic*: fix pH or dynamic pH (default: *fix*)
- optional keyword = *ph* or *buffer*

  ph value = influence ph (default: 7.5)
  buffer value = *phlo phhi* = ph buffer (default: 6.5 9)

Required data sections

`Nutrients`, `Charge Number` sections.

Examples

```
fix kge all kinetics/ph fix
fix kge all kinetics/ph dynamics ph 7.5 buffer 6 8
```

Description

Perform the calculations of pH dynamics and concentrations of nutrient dissociation forms in the liquid solution. A Newton-Raphson implicit method is implemented to find the root of the following equation:

$$[H+] - [OH^-] = \sum_{j}^{i=1} z[S^{z-}] \quad (1)$$

where j is the total number of all the dissociation forms contributing to the pH, z is the number of charge of each form defined in `Charge Number` section, C^{z-} is the concentration of each form calculated based on Gibbs free energy and temperature.

If the keyword *fix* is defined, then we assume pH is constant everywhere in the computational domain, while using *dynamic* will solve the pH based on the above equation.

The key keyword *buffer* will buffer the pH in bulk liquid and maintain the value between the range *phlo phhi*. Two additional chemical species (Na and CL) must be defined in the `Nutrients` section if the keyword is used.

4.8 fix kinetics/thermo command

Syntax

```
fix ID group-ID kinetics/thermo keyword value
```

- *ID* : user-assigned name for the fix
- *group-ID* : ID of the group of microbes to apply the fix to
- *kinetics/thermo* : style name of this fix command
- optional keyword = *reactor* or *yield* or *pressure*

  yield value = *fix* or *dynamic* = flag of dynamic yield (default: *fix*)
  reactor value = *close* or *open* = flag of reactor state (default: *open*)
  gvol value = gas volume ($8e^{-14}$ L)
  rg value = universal gas constant for gas transfer(0.08205746 atm L mol^{-1} K^{-1})

Required data sections

`Atoms`, `Nutrients`, `Catabolism Coeffs`, `Anabolism Coeffs`, `Nutrient Activity Coeffs`, `Type Activity Coeffs`, `Dissipation`*, `Mass Transfer Coeffs`**, `Electron Donor`* sections.

$*$ If *yield-flag* = *dynamic*.
$**$ If *reactor-flag* = *close*.

Examples

```
fix kge all kinetics/thermo
fix kge all kinetics/thermo yield dynamic reactor close
```

Description

Calculate Gibbs free energy and liquid-gas transfer that effect the energy-based growth kinetics. The Gibbs free energy of catabolism ΔG_{cat} and anabolism ΔG_{ana} is given by:

$$\Delta G = \Delta G_0 + R_{th} T (M^T ln(S_{liq}))$$

where R_{th} and T are the universal gas constant and temperature defined in `fix kinetics` command respectively. M is the catabolism and anabolism coefficients derived from `Catabolism Coeffs` and `Anabolism Coeffs` sections, and S_{liq} is the concentrations of nutrient dissociation forms calculated in `fix kinetics/ph` command.

If *yield* = *dymanic*, a dynamic yield value will be calculated at each grid cell. The growth yield is calculated assuming that the Gibbs energy values of the anabolic and catabolic equations plus an energy dissipation term give a fair approximation:

$$Y = -\frac{\Delta G_{cat}}{\Delta G_{ana} + \Delta G_{dis}} + eD$$

where ΔG_{dis} is the dissipation constant defined in `Dissipation` section and eD is electron donor defined in `Electron Donor` section.

Some of the soluble components might transfer in an important proportion to the gas phase. The equilibrium between the liquid and the gas, disturbed by the acid-base reactions and the bacterial activity occurring in the liquid, could move towards production and/or consumption of mass that would affect the reaction term when solving diffusion-reaction. Flag *reactor = close* triggers the gas field to be considered in the simulation domain. To solve gas-liquid transfer of any chemical component, both of its liquid and gas status must be given in `Nutrients` section. For example, if liquid dioxide is named as 'co2', then its corresponding gas status 'gco2' must be defined in order to solve for their gas-liquid transfer.

The reaction rate from gas to liquid $R_{G\rightarrow L}$ of a given nutrient can be expressed by the following equation :

$$R_{G\rightarrow L} = K_L a \cdot (S_{gas} - \frac{S_{liq}}{K_H})$$

here the rate is determined by its mass transfer coefficient $K_L a$ defined in `Mass Transfer Coeffs` section, the gas concentration S_{gas} in the head space, the concentration of dissociation form S_{liq}, and Henry's constant K_H. The transference from liquid to gas is formalised as follow:

$$R_{L\rightarrow G} = \frac{-R_{G\rightarrow L} V_{gas}}{R_g T}$$

where V_{gas} and R_g are the gas volume of the head space (*gvol*), and ideal gas constant (*rg*) defined in the command respectively.

4.9 fix kinetics/diffusion command

Syntax

```
fix ID group-ID kinetics/diffusion v_tol xbc ybc zbc unit
```

- *ID* : user-assigned name for the fix
- *group-ID* : ID of the group of microbes to apply the fix to
- *kinetics/diffusion* : style name of this fix command
- *v_tol* : absolute tolerance to detect convergence
- *xbc, ybc, zbc*: boundary condition mode flag on x, y and z planes (*xbc = ybc = zbc = nn or nd or pp or dn or dd*)
- *unit*: unit used in the command (*units = mol or kg*)
- optional keyword = *bulk* or *srate*

  bulk values = q rvol af
    q = volumetric flow rate (m^3 s^{-1})
    rvol = volume of biofilm reactor (m^3)
    af = total biofilm area in the reactor (m^2)
  srate value = effects of shear rate on the diffusion

Required data sections

`Nutrients`, `Diffusion Coeffs` sections.

Examples

```
variable tol equal 1e-6
...
fix kd all kinetics/diffusion v_tol pp pp nd mol
fix kd all kinetics/diffusion v_tol pp pp nn kg srate 1.0
fix kd all kinetics/diffusion v_tol pp pp nn kg bulk 2.31e-7 1.25e-3 0.1
```

Description

Solve mass balance of soluble substrates in biofilm and bulk liquid. Nutrient distribution within the rectangular simulation domain is calculated by solving the advection-diffusion-reaction equation for each soluble component defined in `Nutrients` section. For each nutrient, the mass balance equation is given by,

$$\frac{\partial S}{\partial t} = \nabla \cdot (D_e \nabla S) - \overrightarrow{U} \cdot \nabla S + R$$

where R is nutrient consumption rate calculated in `kinetics/growth/monod` or `kinetics/growth/energy` and/or `fix kinetics/thermo` command and D is the effective diffusion coefficient defined in `Diffusion Coeffs` section. The equation is discretized on a Marker-And-Cell (MAC) uniform grids defined by *nx*, *ny* and *nz* in `kinetics` command. The temporal and spatial derivatives of the transport equation are discretized by Forward Euler and Central Finite Differences, respectively. This equation is solved for the steady state solution of the concentration fields which is governed by *v_tol*. Six boundary conditions have been implemented in the function, where n = Neumann, d = Dirichlet and p = Periodic. *zbc = nd* means Neumann in *zlo* surface and Dirichlet in *zhi* surface, and same for *xbc* and *ybc*.

unit defines concentration unit used during the computation. If `kinetics/growth/energy` command is used, then *unit* has to be set as *mol*. If `kinetics/growth/monod` command is used, then *unit* has to be set as *kg*.

If the optional keyword *bulk* is defined in the command, the function will solve the mass balances of solutes in the bulk liquid for dynamic conditions:

$$\frac{dS^{(b)}}{dt} = \frac{Q}{V}(S^{(in)} - S^{(out)}) + \frac{A_f}{VL_YL_Z}\int_0^{L_X}\int_0^{L_Y}\int_0^{L_Z} R(x,y,z)dzdydx$$

where Q is the volumetric flow rate (q), V is the volume of biofilm reactor (rvol), A_f is the total biofilm area in the reactor (af) and L is the size of simulation domain. The equation updates the concentration in *zhi* surface at each kinetics step.

4.10 divide command

Syntax

```
fix ID group-ID divide Nevery v_EPSdens v_divMass seed
```

- *ID* : user-assigned name for the fix
- *group-ID* : ID of the group of microbes to apply the fix to
- *divide* : style name of this fix command
- *Nevery* : call the function every this many timesteps
- *v_EPSdens* : density of EPS (kg m^{-3})
- *v_divDia* : threshold mass value for microbe division (kg)
- *seed* : random seed for cell orientation
- optional keyword = *demflag*

    demflag value = 0 or 1 = flag of DEM run (default: 0)

Required data sections

`Atoms` section

Examples

```
variable EPSdens equal 30
variable divDia equal 1.3e-6

fix d1 all divide 500 v_EPSdens v_divDia 111
```

Description

Perform division for the microbes in group. The function is implemented in following way: If the mass of a microbe becomes greater than a user-defined value (which is normally twice the mass of an inoculated individual bacterium), it divides into two daughter cells each. During the division process, the cell mass is split in a ratio randomly selected between 0.4-0.6. This generated two daughter cells from a parent cell. These daughter cells are oriented randomly around the centre of the parent cell.

The *v_EPSdens* setting is required for the divisions of HET particles. Value can be floating type number.

The *v_divDia* parameter defines the threshold diameter value that a microbe starts dividing.

4.11 eps_extract command

Syntax

```
fix ID group-ID eps_extract Nevery v_EPSratio v_EPSdens seed
```

- *ID* : user-assigned name for the fix
- *group-ID* : ID of the group of microbes to apply the fix to
- *eps_extract* : style name of this fix command
- *Nevery* : call the function every this many timesteps
- *v_EPSratio* : extraction threshold ratio between outer-radius and inner-radius of HET
- *v_EPSdens* : density of EPS
- *seed* : random seed for cell orientation
- optional keyword = *demflag*

  demflag value = 0 or 1 = flag of DEM run (default 0)

Required data sections

`Atoms` section

Example

```
variable v_EPSratio equal 1.25
variable v_EPSdens equal 30

fix d1 HET eps_extract 500 v_EPSratio v_EPSdens 123
```

Description

Perform EPS production process in the simulation. To use the command, a species named "eps" must be defined in `Atoms` section.

Microbes secrete extracellular polymeric substances (EPS) every so often as a waste product of their metabolic activities. EPS is secreted into their neighbouring environment and have known to lend structural integrity to the biofilms. The implementation works on the common knowledge that HETs excrete EPS, while other microbial species do not. Initially, EPS is accumulated as a extra shell beyond the HET particle. It should be noted that the EPS density is much lower than the HET density. When the relative thickness of the EPS shell bound to HET particle exceeds a certain threshold value, i.e., *v_EPSratio* value, almost half (random ratio between 0.4-0.6) of the EPS mass excretes as a separate EPS particle and positions next to the HET cell.

4.12 death command

Syntax

```
fix ID group-ID death Nevery v_deadDia
```

- *ID* : user-assigned name for the fix
- *group-ID* : ID of the group of microbes to apply the fix to
- *death* : style name of this fix command
- *Nevery* : call the function every this many timesteps
- *v_deadDia* : threshold diameter value for microbe death

Required data sections

`Atoms` section

Example

```
variable v_deadDia equal 0.8e-6

fix d1 HET death 500 v_deadDia
```

Description

Perform microbe death process in the simulation. To use the command, a species named "dead" must be defined in `Atoms` section.

The size of microbe decreases when there is not enough nutrient to uptake. Microbe dies if a threshold is reached. The *v_deadDia* defines how small a microbe may become before changing the type to DEAD. We assume that there is no biological activity in dead microbes and their sizes remain unchanged.

4.13 epsadh command

Syntax

```
fix ID group-ID epsadh Nevery v_ke f_adhmodel
```

- *ID* : user-assigned name for the fix
- *group-ID* : ID of the group of microbes to apply the fix to
- *epsadh* : style name of this fix command
- *Nevery* : call the function every this many timesteps
- *v_ke* : spring stiffness
- *f_adhmodel* : adhesive force model flag (*f_adhmodel = 1 or 2*)

Required data sections

`Atoms` section

Example

```
variable ke equal 5e+10

fix d1 HET epsadh 1 v_ke 1
```

Description

The excreted EPS mass from the HET particles can be employed as a parameter of adhesion force models between the particles. The EPS link between the particles are treated as much more stiffer springs, but only employing the attractive forces. Total effective EPS mass M_{ij}^{eps} is calculated between the microbes, and a spring stiffness k_e is defined per unit mass. The forces calculated according to the effective spring stiffness ($M_{ij}^{eps}k_e$) multiplied by the separation distance between two particles (model 1), or inverse of the separation distance (model 2).

The EPS-mediated binding forces are calculated as:

$$\overrightarrow{F}_{eps,ij} = M_{ij}^{eps} k_e (d_{ij} - d_{0ij}) \cdot \frac{\overrightarrow{d}_{ij}}{d_{ij}}$$

$$\overrightarrow{F}_{a,i} = \sum_{j=1}^{N} \overrightarrow{F}_{eps,ij}$$

where d_{0ij} is the sum of the radii of two interacting particles and d_{ij} is the distance between centres of two particles.

4.14 walladh command

Syntax

```
fix ID group-ID walladh v_kanc f_wallstyle lo hi
```

- *ID* : user-assigned name for the fix
- *group-ID* : ID of the group of microbes to apply the fix to
- *walladh* : style name of this fix command
- *v_kanc* : adhesive strength
- *f_wallstyle* : specify a pair of walls in a dimension (*f_wallstyle = xplane or yplane or zplane*)
- *lo, hi* : position of lower and upper plane

Required data sections

`Atoms` section

Examples

```
variable kanc equal 50

fix xw all walladh v_kanc xplane 0.0 1.0e-04
fix yw all walladh v_kanc yplane 0.0 5.0e-05
```

Description

Impose an adhesive force between the wall (the boundary of simulation domain) and the microbes attaching to the wall. The force is calculated as the product of adhesive strength and overlap distance.

4.15 shear command

Syntax

```
fix ID group-ID shear Nevery v_viscosity v_shearRate v_height
f_direction start end
```

- *ID* : user-assigned name for the fix
- *group-ID* : ID of the group of microbes to apply the fix to
- *shear* : style name of this fix command
- *Nevery* : call the function every this many timesteps
- *v_viscosity*: dynamic viscosity of fluid
- *v_shear-rate*: rate of change of velocity
- *v_height* : distance to the stationary point from bottom wall
- *f_direction* : direction of the force (*direction = zx or zy*)
- *start, end* : time range for applying the force

Required data sections

`Atoms` section

Examples

```
variable viscosity equal 0.5
variable shearRate equal 0.6
variable height equal 5e-5
fix s1 all shear 10 v_viscosity v_shearRate v_height zx 5 500
```

Description

Impose an additional shear force each microbe in the group. The shear force is calculated according to the drag force created on a sphere in Stokes flow, and it is given by:

$$\overrightarrow{F}_{f,i} = 6\pi\mu r_i \overrightarrow{v_r}$$

where μ is dynamic viscosity of fluid, r_i is radius of particle and $\overrightarrow{v_r}$ is local fluid velocity relative to the particle. The parameter *height* is a user-defined value where the directions of flow above and blow the stationary point are in opposition.

4.16 cfddem command (Sedifoam thirdparty)

Syntax

```
fix ID group-ID cfddem v_demSteps v_bioSteps v_bioDt v_nloops
```

- *ID* : user-assigned name for the fix
- *group-ID* : ID of the group of microbes to apply the fix to
- *cfddem* : style name of this fix command
- *v_demSteps*: # of mechanical (DEM) steps between two fluid tiemsteps
- *v_bioSteps*: # of biological steps in a single loop
- *v_bioDt* : biological timestep
- *f_nloops* : # of total loops

Examples

```
variable demSteps equal 100
variable bioSteps equal 60
variable bioDt equal 10
variable nloops equal 20

fix cfd all cfddem v_demSteps v_bioSteps v_bioDt v_nloops
```

Description

This fix command defines control parameters used in hydrodynamic biofilm simulation (CFD-DEM). The procedure of OpenFOAM and LAMMPS coupling is shown in Algorithm 2.

Algorithm 2 (kinetics integration procedure)

```
i := 0;
while i < nloops do
    perform n = v_bioSteps biological steps with timestep = bioDt (solving growth,
    division, diffusion etc)
    j:= 0
    while j < Fluid_EndTime do
        solve fluid, transfer information from OpenFOAM to LAMMPS
        preform m = v_demSteps DEM steps with timestep = demDt in LAMMPS
        transfer information from LAMMPS to OpenFOAM
        j += fluidDt
    end while
    i++
end while
```

Here, the `v_bioSteps`, `v_demSteps`, `nloops` and `bioDt` are defined in this command. The DEM timestep `demDt` is defined in LAMMPS `timestep` command. The fluid timestep `fluidDt` and fluid end time `Fluid_EndTime` are defined in OpenFOAM input files, see `https://www.openfoam.com/documentation/` for more details.

4.17 compute ntypes command

Syntax

```
compute ID group-ID ntype
```

- *ID* : user-assigned name for the computation
- *group-ID* : ID of the group of atoms to perform the computation on
- *ntype* : style name of this compute command

Examples

```
compute myNtypes all ntypes
```

Description

Define a computation that calculates total number of microbes of each species in the system. Result values are saved in a global vector that can be output via `dump bio` or `thermo_style` command.

4.18 compute biomass command

Syntax

```
compute ID group-ID biomass
```

- *ID* : user-assigned name for the computation
- *group-ID* : ID of the group of atoms to perform the computation on
- *biomass* : style name of this compute command

Examples

```
compute myMass all biomass
```

Description

Define a computation that calculates total biomass of each species in the system. Result values are saved in a global vector that can be output via `dump bio` or `thermo_style` command.

4.19 compute diameter command

Syntax

```
compute ID group-ID diameter
```

- *ID* : user-assigned name for the computation
- *group-ID* : ID of the group of atoms to perform the computation on
- *diameter* : style name of this compute command

Examples

```
compute myDia all diameter
```

Description

Define a computation that calculates floc equivalent diameter. The specified group must be "all". The equivalent diameter at time t $d_{t,eqv}$ is computed by the formula:

$$d_{t,eqv} = \sum_{k=1}^{n} \sqrt[3]{\frac{6V_{kt}}{\pi}}$$

where V_{kt} is volume of each individual spherical particle k at time t. Result value is saved in a global scalar that can be output via `dump bio` or `thermo_style` command.

4.20 compute dimension command

Syntax

```
compute ID group-ID dimension
```

- *ID* : user-assigned name for the computation
- *group-ID* : ID of the group of atoms to perform the computation on
- *dimension* : style name of this compute command

Examples

```
compute myDimen all dimension
```

Description

Define a computation that calculates fractal dimension. The specified group must be "all". The fractal dimension F_{Dt} is computed by the formula:

$$F_{Dt} = \frac{log(R_a/R_m)}{log(n)}$$

where n is total number of particles in the system, $R_a = \sqrt{\frac{\sum_{k=1}^{n} m_k d_k^2}{\sum_{k=1}^{n} m_k}}$ and $R_m = \frac{\sum_{k=1}^{n} r_k}{n}$, d_k, r_k and m_k are the particle diameter, radius and mass respectively. Result value is saved in a global scalar that can be output via `dump bio` or `thermo_style` command.

4.21 compute diversity command

Syntax

```
compute ID group-ID diversity
```

- *ID* : user-assigned name for the computation
- *group-ID* : ID of the group of atoms to perform the computation on
- *diversity* : style name of this compute command

Examples

```
compute myDiver all diversity
```

Description

Define a computation that calculates diversity index in a system. The specified group must be “all”. The diversity index D_t at time t is computed by the formula:

$$D_t = 1 - \frac{\sum n(n-1)}{N(N-1)}$$

Where n is the total number of organism of a particular specie and N is the total number of microbes of all species. Result value is saved in a global scalar that can be output via `dump bio` or `thermo_style` command.

4.22 compute ave_height command

Syntax

```
compute ID group-ID ave_height nx ny
```

- *ID* : user-assigned name for the computation
- *group-ID* : ID of the group of atoms to perform the computation on
- *ave_height* : style name of this compute command
- optional keyword = *nx ny*

  nx value *ny* value = number of grid elements in x, y planes

Examples

```
compute myHeight all ave_height
compute myHeight all ave_height nx 50 ny 50
```

Description

Define a computation that calculates biofilm average height. The specified group must be “all”. The formula is given by:

$$\overline{h} = \frac{1}{L_x L_y} \int \int h(x,y) dxdy$$

where $h(x,y)$ is biofilm height (measured in z direction) at position (x,y) on the substratum. By default, the simulation domain is discretized automatically where the size of each grid element is the maximum cut-off distance for all atom pairs used in mechanical iteration. Result value is saved in a global scalar that can be output via `dump bio` or `thermo_style` command.

4.23 compute roughness command

Syntax

```
compute ID group-ID roughness nx ny
```

- *ID* : user-assigned name for the computation
- *group-ID* : ID of the group of atoms to perform the computation on
- *roughness* : style name of this compute command
- optional keyword = *nx ny*

  nx value *ny* value = number of grid elements in x, y planes

Examples

```
compute myRough all roughness
compute myRough all roughness nx 50 ny 50
```

Description

Define a computation that calculates biofilm roughness. The specified group must be "all". The formula is given by:

$$roughness = \frac{1}{L_x L_y} \int \int (h(x,y) - \overline{h})^2 dxdy$$

where $h(x,y)$ is biofilm height (measured in z direction) at position (x,y) on the substratum. $\overline{h}$ is biofilm average height. By default, the simulation domain is discretized automatically where the size of each grid element is the maximum cut-off distance for all atom pairs used in mechanical iteration. Result value is saved in a global scalar that can be output via `dump bio` or `thermo_style` command.

4.24 compute segregation command

Syntax

```
compute mySeg all segregation v_cutoff
```

- *ID* : user-assigned name for the computation
- *group-ID* : ID of the group of atoms to perform the computation on
- *segregation* : style name of this compute command
- *v_cutoff* : cutoff distance for neighbour list calculation (m)

Examples

```
variable cutoff equal 1e-6

compute mySeg all segregation v_cutoff
```

Description

Define a computation that calculates biofilm segregation index. The specified group must be “all”. The formula is given by:

$$\sigma_t = \frac{1}{M}\sum_{i=1}^{M}(\frac{1}{N}\sum_{j=1}^{N}\rho(c_i, c_j))$$

$$\text{where } \rho(c_i, c_j) = \begin{cases} 0, c_j \text{ is not the same type as } c_i \\ 1, c_j \text{ is the same type as } c_i \end{cases}$$

j is neighbour of atom *i*, variable *v_cutoff* defines neighbour list of each atom. Result value is saved in a global scalar that can be output via `dump bio` or `thermo_style` command.

4.25 dump bio command

Syntax

```
dump ID group-ID bio Nevery file args
```

- *ID* : user-assigned name for the dump
- *group-ID* : ID of the group of atoms to be dumped
- *bio* : style name of this dump command
- *Nevery* : dump every this many timesteps
- *file* : name of file to write dump info to
- *args* : list of arguments for dump info

```
possible dump info = biomass, ntypes, diameter, dimension,
                     diversity, ave_height, roughness,
                     segregation, avg_con, avg_ph, bulk
```

```
biomass = total biomass of each species
ntypes = total number of microbes of each species
diameter = floc equivalent diameter
dimension = fractal dimension
diversity = diversity index
avg_height = biofilm average height
roughness = biofilm roughness
segregation = segregation index
avg_con = average nutrient concentration
bulk = nutrient concentration in bulk liquid
avg_ph = average pH
```

Examples

```
compute myMass all biomass
compute myRough all roughness
dump d0 all bio 1000 biomass roughness
```

Dump IBm attributes to one or more data files every *Nevery* timesteps. The related computes of specified attributes must be defined in prior. The dumped attribute information are related to biofilm and floc. To dump atom information (e.g, mass, diameter, position), use `dump custom` command, or `dump custom/vtk` command. The `dump bio` command creates a new directory named `/Result` in the current example directory to save the output data.

4.26 dump grid command

Syntax

```
dump ID group-ID grid Nevery file args
```

- *ID* : user-assigned name for the dump
- *group-ID* : ID of the group of atoms to be dumped
- *grid* : style name of this dump command
- *Nevery* : dump every this many timesteps
- *file* : name of file to write dump info to
- *args* : list of arguments for dump info

```
possible dump info = con, upt, gas, act, yie, cat, ana
```

```
con = nutrient concentration
upt = nutrient update rate
gas = gas field
act = nutrient activity
yie = growth yield
cat = Gibbs free energy of anabolism
ana = Gibbs free energy of catabolism
```

Examples

```
dump dv all grid 10000 grid_%_*.vti con
dump dv all grid 500 grid_%_*.vti yie cat
```

Dump a snapshot of field information to one or more files every N timesteps in a format readable by the VTK visualization toolkit or other visualization tools that use it, e.g. ParaView.

Similarly with `dump custom/vtk` command. The VTK format uses a single snapshot of the system per file, thus a wildcard "*" must be included in the filename, as discussed below. Otherwise the dump files will get overwritten with the new snapshot each time.

Dump filenames can contain two wildcard characters. If a "*" character appears in the filename, then one file per snapshot is written and the "*" character is replaced with the timestep value. For example, grid*.vti becomes grid0.vti, grid10000.vti, grid20000.vti, etc.

If a "%" character appears in the filename, then each of P processors writes a portion of the dump file, and the "%" character is replaced with the processor ID from 0 to P-1 preceded by an underscore character. For example, grid%.vti becomes grid0.vti, grid1.vti, . . . gridP-1.vti, etc. This creates smaller files and can be a fast mode of output on parallel machines that support parallel I/O for output.

4.27 dump bio/hdf5 command

Syntax

```
dump ID group-ID bio/hdf5 Nevery file args
```

- *ID* : user-assigned name for the dump
- *group-ID* : ID of the group of atoms to be dumped
- *bio/hdf5* : style name of this dump command
- *Nevery* : dump every this many timesteps
- *file* : name of file to write dump info to
- *args* : list of arguments for dump info

```
possible dump info = id, type, x, y, z, vx, vy, vz, fx, fy, fz,
                     radius, con, upt, gas, act, yie, cat, ana
```

```
id = atom id
type = atom type
x, y, z = atom position
vx, vy, vz = atom velocity
fx, fy, fz = atom force
radius = atom radius
con = nutrient concentration
upt = nutrient update rate
gas = gas field
act = nutrient activity
yie = growth yield
cat = Gibbs free energy of anabolism
ana = Gibbs free energy of catabolism
```

Examples

```
dump dh0 all bio/hdf5 3600 dump_*.h5 id type radius x y z con cat ana hyd
```

Dump filenames can contain a wildcard character. If a “*” character appears in the filename, then one file per snapshot is written and the “*” character is replaced with the timestep value. For example, grid*.vti becomes grid0.vti, grid10000.vti, grid20000.vti, etc.

5 The NUFEB developer team

- Bowen Li (bowen.li2@newcastle.ac.uk)
  - Software Developer
- Denis Taniguchi (denis.taniguchi@newcastle.ac.uk)
  - Software Developer
- Jayathilake Pahala Gedara (pgjayathilake@gmail.com)
  - Biomechanical modelling
- Rebeca Gonzalez Cabaleiro (rebeca.gonzalez-cabaleiro@glasgow.ac.uk)
  - Biochemical modelling
- Valentina Gogulancea (valentina.gogulancea@newcastle.ac.uk)
  - Biochemical modelling

A Define a Multi-loops run

The time scale of biological processes ($\approx$ 1 hour) is in general much slower than the mechanical ($\approx 10^{-8}$ hours) and chemical processes ($\approx 10^{-6}$ hours). Thus, the later two can be treated as instantaneous processes without considering biological activities. To solve the system, an IBm usually separates the processes. The profiles of the dissolved components and the mechanical interaction between microbes can be calculated by a steady-state models. While the biofilm itself is modeled as dynamic. After each biological timestep, when the shape and composition of the biofilm have changed enough, the steady state distribution of the dissolved components and mechanical interaction have to be recalculated.

In NUFEB, a general way to model the above system is to use multi-loops simulation scheme. A main loop with larger time-scale is defined to perform biological activities. At each timestep of the main loop, two sub-loops are defined to solve chemical and mechanical processes until they reach a pseudo-steady state or certain maximum steps. An example is the kreft-energy cases located in `NUFEB/example/kreft-energy` directory. The inputscript is something like:

```
...
pair_style  gran/hooke/history 1.e-4 NULL 1.e-5 NULL 0.0 1
...
fix k1 all kinetics 1 100 5 100 v_diffT v_layer demflag 0
fix d1 all divide 1 v_EPSdens v_divDia 31231 demflag 0
...
#############Define Bio-loop and DEM-subloop#############

run 4320 pre no post no every 1 &
"fix_modify k1 demflag 1" &
"fix_modify d1 demflag 1" &
"timestep 1e-3" &
"run 10000 pre no post no" &
"timestep 600" &
"fix_modify k1 demflag 0" &
"fix_modify d1 demflag 0" &
```

The main (biological) loop has 4320 steps. The *every* keyword is used to break a NUFEB run into a series of shorter runs where the sub-loops are inserted. The *pre* and *post* keywords are used to streamline the setup, clean-up, and associated output to the screen that happens before and after each shorter run. In this case, they should be specified as “no” so the initial setup and full timing summary are skipped.

At each shorter run (biological step), the simulation will first proceed chemical and biological processes (e.g, fix kinetics, and fix divide). Then these processes will be switched off by setting *demflag = 1* via `fix modify` command. This will make sure that the chemical and biological processes will not proceed during mechanical sub-loop. Then we change to the mechanical timestep (1e-3 s) and perform a 10000 steps sub-simulation for the mechanical interaction (e.g, impose frictional force by gran/hooke/history). After the mechanical loop, we reset the timestep to biological timescale and also switch on chemical and biological processes ready for the next step by setting *demflag = 0*.
